# Massive contractions of myotonic dystrophy type 2-associated CCTG tetranucleotide repeats occur via double-strand break repair with distinct requirements for DNA helicases

**DOI:** 10.1093/g3journal/jkad257

**Published:** 2023-11-10

**Authors:** David Papp, Luis A Hernandez, Theresa A Mai, Terrance J Haanen, Meghan A O’Donnell, Ariel T Duran, Sophia M Hernandez, Jenni E Narvanto, Berenice Arguello, Marvin O Onwukwe, Sergei M Mirkin, Jane C Kim

**Affiliations:** Department of Biological Sciences, California State University San Marcos, San Marcos, CA 92078, USA; Department of Biological Sciences, California State University San Marcos, San Marcos, CA 92078, USA; Department of Biological Sciences, California State University San Marcos, San Marcos, CA 92078, USA; Department of Biological Sciences, California State University San Marcos, San Marcos, CA 92078, USA; Department of Biological Sciences, California State University San Marcos, San Marcos, CA 92078, USA; Department of Biological Sciences, California State University San Marcos, San Marcos, CA 92078, USA; Department of Biological Sciences, California State University San Marcos, San Marcos, CA 92078, USA; Department of Biological Sciences, California State University San Marcos, San Marcos, CA 92078, USA; Department of Biological Sciences, California State University San Marcos, San Marcos, CA 92078, USA; Department of Biological Sciences, California State University San Marcos, San Marcos, CA 92078, USA; Department of Biology, Tufts University, Medford, MA 02155, USA; Department of Biological Sciences, California State University San Marcos, San Marcos, CA 92078, USA

**Keywords:** DNA repeats, microsatellites, DNA repair, homologous recombination

## Abstract

Myotonic dystrophy type 2 (DM2) is a genetic disease caused by expanded CCTG DNA repeats in the first intron of *CNBP*. The number of CCTG repeats in DM2 patients ranges from 75 to 11,000, yet little is known about the molecular mechanisms responsible for repeat expansions or contractions. We developed an experimental system in *Saccharomyces cerevisiae* that enables the selection of large-scale contractions of (CCTG)_100_ within the intron of a reporter gene and subsequent genetic analysis. Contractions exceeded 80 repeat units, causing the final repetitive tract to be well below the threshold for disease. We found that Rad51 and Rad52 are involved in these massive contractions, indicating a mechanism that uses homologous recombination. Srs2 helicase was shown previously to stabilize CTG, CAG, and CGG repeats. Loss of Srs2 did not significantly affect CCTG contraction rates in unperturbed conditions. In contrast, loss of the RecQ helicase Sgs1 resulted in a 6-fold decrease in contraction rate with specific evidence that helicase activity is required for large-scale contractions. Using a genetic assay to evaluate chromosome arm loss, we determined that CCTG and reverse complementary CAGG repeats elevate the rate of chromosomal fragility compared to a short-track control. Overall, our results demonstrate that the genetic control of CCTG repeat contractions is notably distinct among disease-causing microsatellite repeat sequences.

## Introduction

Repeat expansion diseases are caused by excessively long tracts of simple DNA sequence repeats, also called microsatellites, within specific genes. Two types of myotonic dystrophy (DM) are examples of such diseases (Meola and Cardani 2015). DM1 is caused by long (>50) CTG trinucleotide repeats (TNRs) in the 3′ UTR of *DMPK*, whereas DM2 is caused by long (>75) tetranucleotide CCTG repeats in the first intron of *CNBP*. Transcription of these expanded alleles results in cellular toxicity through RNA gain-of-function effects (Sznajder and Swanson 2019). Repeat-associated non-ATG translation products were observed in brain tissues of DM1 and DM2 patients (Zu *et al*. 2011, 2017), suggesting that aberrant proteins may also contribute to pathophysiology. Disease symptoms include the inability to relax muscles after they have contracted, muscle weakness, and cardiac conduction problems. However, no cure or specific therapy is currently available for either disease, with treatment relying on symptom management.

Individuals with DM1 or DM2 may have thousands of tandem repeats within the affected gene. Through studies using model organisms and cell culture systems, there have been substantial advances in understanding CTG and reverse complementary CAG instability (expansions and contractions) (Usdin *et al*. 2015; Polyzos and McMurray 2017; Khristich and Mirkin 2020; Wheeler and Dion 2021). CAG/CTG repeats form stable hairpins and slipped strands in vitro, and the formation of these secondary structures in vivo is proposed to instigate various molecular mechanisms of instability (Pearson and Sinden 1998; Kim and Mirkin 2013; Poggi and Richard 2021). For example, polymerase slippage followed by hairpin formation on the nascent strand during DNA replication or repair synthesis would lead to an expansion. In contrast, structure formation on the template strand, which is then bypassed during DNA synthesis, would result in repeat contractions. Additionally, CAG/CTG repeats cause replication fork stalling (Fouche *et al*. 2006; Kerrest *et al*. 2009) and elevated DNA double-strand break (DSB) formation (Callahan *et al*. 2003; Polleys and Freudenreich 2020), and their recovery or repair via homologous recombination (HR) could lead to expansions or contractions depending on the fidelity of steps such as strand annealing (Polleys and Freudenreich 2021).

Understanding the molecular mechanisms of contractions is important to evaluate whether manipulating this process would be a viable approach to treat repeat expansion diseases (Izzo *et al*. 2022). Strategies to induce targeted repeat contractions using either small molecules that specifically bind to slipped strand intermediates (Nakamori *et al*. 2020) or sequence-specific endonucleases to generate DSBs at the repeat locus (Cinesi *et al*. 2016) were successful in experimental systems with CAG/CTG repeats. It is unclear whether these strategies would apply generally to all disease-causing DNA repeat sequences or whether the underlying mechanisms of large-scale contractions for different repeats are also distinct. Specifically, we designate the descriptors massive or large scale as the number of repeats that would result in a decrease from symptomatic to unaffected lengths (such as a deletion in >45 repeats for DM2 as opposed to loss of just a few repeats).

In contrast to CAG/CTG repeats, much less is known about the mechanisms of CCTG and reverse complementary CAGG repeat instability. This is a pressing question since individuals with DM2 can have 75–11,000 CCTG repeats in *CNBP* with an estimated average of 5,000 repeats (Liquori *et al*. 2001). DM2 is also the only repeat expansion disease currently known to be caused by tetranucleotide repeats. In vitro studies with chemical and enzymatic probing showed that CAGG repeats form stable hairpin structures with a specific base-pairing propensity, which was not prominent for the CCTG orientation (Dere *et al*. 2004). CAGG/CCTG repeats were also shown to form slipped-strand structures through denaturation and reduplexing experiments (Edwards, Sirito, *et al*. 2009). In addition, NMR analysis indicated that CCTG repeats form hairpin and dumbbell structures that are much more fluid and dynamic (Lam *et al*. 2011), displaying an ability to change between different conformations and shift along the DNA duplex. This fluidity is proposed to increase the likelihood that CCTG repeats could escape DNA repair (Guo and Lam 2016a; Guo and Lam 2016b).

Previous studies investigating CAGG/CCTG repeat instability in vivo have relied on plasmid-based systems using *Escherichia coli* and COS-7 mammalian cell culture (Dere *et al*. 2004; Dere and Wells 2006). The frequency of expansions and contractions increased with longer starting lengths, and the orientation with CAGG repeats on the leading strand template showed greater instability. Another plasmid study using *E. coli* found that instability was not dependent on repeat orientation (Edwards, Hashem, *et al*. 2009).

In the current study, we developed a yeast experimental system to investigate the instability of CAGG/CCTG repeats within a chromosomal context. This system offers an additional advantage of enabling the selection of repeat contractions that exceed 80 repeats in length, for example, from (CCTG)_100_ to (CCTG)_20_. Through genetic analysis, we found that HR is involved in large-scale CCTG repeat contractions. We also demonstrate that CAGG/CCTG repeats elevate the rate of DSB formation using a genetic assay for chromosomal arm loss. Altogether, genes that have been well-characterized to control CAG/CTG repeat instability do not affect CAGG/CCTG repeats in the same manner, indicating that the precise genetic control of DNA repeat instability is unique to the specific microsatellite sequence.

## Materials and methods

### CAGG/CCTG plasmid cloning

PCR was used to amplify a double-stranded product using oligonucleotides with complementary CCTG and CAGG repeats that anneal at their 3′ ends. JK178 has a 5′ NcoI site followed by CCTG repeats, and JK179 has a 5′ SphI site followed by CAGG repeats (Supplementary Table 1). The amplified product was digested with NcoI and SphI and then cloned into the plasmid designated pYes3-G4G1C1-T150-GAA100 that was digested with the same enzymes (Shah *et al*. 2014). A plasmid containing (CCTG)_27_ was verified by Sanger sequencing. Long CCTG repeats were cloned using CCTG and CAGG oligonucleotides designed with 5′ nonpalindromic restriction sites, following a previously described strategy to clone TNRs (Grabczyk and Usdin 1999; Krasilnikova and Mirkin 2004). The repeat tract was amplified from this plasmid using JK188 and JK189, which contain nonpalindromic BsgI sites at their 5′ ends (Supplementary Fig. 1). The PCR product was purified and digested with BsgI (NEB R0559). The (CCTG)n/(CAGG)n repeats are positioned between the 2 inverted BsgI sites in such a way that pure repeats with complementary 3′ TG and CA overhangs are generated upon digestion with BsgI. The digested DNA was purified again and set up in a ligation reaction (NEB M0202). These fragments will ligate in the head-to-tail direction only. The ligated products were separated on an agarose gel, excised, purified, and blunt-ended with T4 DNA polymerase and Klenow fragment. This fragment was cloned into the plasmid backbone described as (GAA)_0_ (Shah *et al*. 2012), which had been blunt-cut with NaeI and treated with alkaline phosphatase. The plasmid was confirmed to have uninterrupted (CCTG)_100_ repeats by Sanger sequencing (Genewiz). The (CCTG)_100_ plasmid is designated p18 and contains an overall intron length of 1,047 bp.

A (CCTG)_100_ plasmid with a shorter overall intron length (820 bp) was obtained by cutting p18 with blunt end restriction enzymes BsaBI and MscI to remove 227 bp of nonrepetitive sequence. The (CAGG)_100_ reverse orientation was obtained through molecular cloning whereby PCR using primers JK354 and JK355 with the p18 template will reverse the position of the XhoI and NotI sites flanking the repeats. The digested fragment was then cloned into p18 that had also been digested with XhoI and NotI.

### Yeast strain construction for large-scale contractions

All strains are isogenic to *Saccharomyces cerevisiae* wild-type (WT) strain CH1585 (*MAT*a, *leu2-Δ1*, *trp1-Δ63*, *ura3–52*, and *his3–200*), an S288C-derived haploid strain used in previous DNA repeat instability studies (ATCC 96098). The reporter cassette was excised from each plasmid using SwaI. Transformants were selected on synthetic complete media lacking tryptophan. The cassette is positioned ∼1-kb downstream of *ARS306*, replacing SGD coordinates 75594–75641 on chromosome III. Correct integration was verified by PCR. The integrity of CCTG repeat length was verified by PCR and Sanger sequencing (Retrogen), and this starting strain is designated YJK168 (Supplementary Table 2).

Except as noted below, gene knockouts and point mutations were constructed using a CRISPR approach with the bRA89 (*HPH*) or bRA90 plasmid (*LEU2*) (Anand *et al*. 2017). The repair template for knockouts was oligonucleotides consisting of the first 45 nucleotides of the open reading frame followed by the last 45 nucleotides (Supplementary Table 3). PCR using primers flanking the disrupted region was used to verify the knockout strains, and additional primers (i.e. JK213/214 or JK402/403) were used to confirm that the repeat length had been maintained. For separation-of-function mutants, the 90-bp repair template included both the gRNA target, introducing a silent mutation into the NGG sequence, as well as the specific separation-of-function mutation. Specific mutations were verified by Sanger sequencing (Retrogen). The Rad52-Y33A allele is referred to as Y66A in its initial description (Mortensen *et al*. 2002) according to the primary sequence initially published (Adzuma *et al*. 1984). The first 33 amino acids in the originally published sequence are not included in the protein as the third start codon in the sequence is the one used. For successful transformants, loss of the CRISPR plasmid was verified via plating to ensure that the Cas9 endonuclease is no longer expressed (i.e. sensitivity to hygromycin).

The *pol32Δ*, *rad59Δ*, *sae2Δ*, *mre11Δ*, *rmi1Δ*, and *exo1Δ* strains were constructed using a PCR-based method for direct gene replacement with pAG32 (HphMX4) (Goldstein and McCusker 1999) used as a template for PCR. Correct targeting was verified by PCR with primers flanking the replacement locus and within the HphMX4 cassette.

### Fluctuation analysis to determine large-scale contraction rates

All strains were initially grown as single colonies on rich media (YPD) agar plates supplemented with 50-μg/mL uracil (referred to as DU) for 72 h at 30°C. Whole, individual colonies were suspended in 1000-μL sterile water. To select for URA+ clones, 100 μL of the cell suspension was directly plated on synthetic complete media lacking uracil (SC-URA), which is composed of 2% glucose, 0.67% yeast nitrogen base, 0.2% drop-out mix synthetic minus uracil (US Biological D9536), and 2% agar. The cell suspension was serially diluted to plate on YPD media (100 μL of 10^−5^ dilution) for determination of total cell number. For each experiment, at least 12 independent colonies were analyzed, including 2 independent isolates of each strain/genotype. Colonies growing on DU that had an initial contraction or expansion, assessed via repeat length PCR, were excluded from the analysis. All colonies on SC-URA and YPD were counted at 72 h. Rates and 95% CIs were calculated using the Ma–Sandri–Sarkar maximum likelihood estimator (MSS-MLE) method with correction for plating efficiency determined as *z* − 1/*z*ln(*z*), where *z* is the fraction of the culture analyzed (Rosche and Foster 2000). The average number of viable cells grown on YPD (Nt) was used in all calculations. The web-hosted program FluCalc was used to perform this rate analysis (Radchenko *et al*. 2018). Large-scale contraction rates were considered to be significantly different if 95% CIs for the rate values did not overlap (Foster 2006).

### Single colony PCR to determine repeat length and generate products for sequencing

Genomic DNA was isolated from single colonies using a previously described method (Aksenova *et al*. 2013). Cells were resuspended in 1.5 μL of 0.5 mg/mL lyticase solution [0.9 M sorbitol, 0.1 M EDTA (pH 7.4)], incubated at 37°C for 15 min, and then resuspended in 50 μL of water. Samples were incubated at 100°C for 5 min and centrifuged at 2,500 × *g* for at least 2 min. For PCR analysis of CCTG/CAGG repeat length using primers JK402 and JK403 (441-bp product for (CCTG)_100_), reactions included 1X GC buffer (F519, Thermo Fisher Scientific), 0.2 mM dNTP mix, 0.8 μM of each primer, 3% DMSO, 0.4 units of Phusion Hot Start II DNA polymerase (F549, Thermo Fisher Scientific), and 1.5 μL of the DNA supernatant in a 20-μL total reaction volume. Thermocycling conditions were 30 s at 98°C, followed by 32 cycles of 10 s at 98°C, 20 s at 60°C, and 30 s at 72°C, concluding with 10 min at 72°C.

For PCR analysis of CCTG/CAGG repeat length using primers JK213 and JK214 (590-bp product for (CCTG)_100_), reactions included 1X Green GoTaq reaction buffer (M7911, Promega), 0.2 mM dNTP mix, 0.8 μM of each primer, 3% DMSO, 1 unit of Taq (Thermo Fisher Scientific EP0402), and 1.5 μL of the DNA supernatant in a 20-μL total reaction volume. Thermocycling conditions were 60 s at 95°C, followed by 32 cycles of 30 s at 95°C, 20 s at 60°C, and 60 s at 72°C, concluding with 10 min at 72°C.

For PCR analysis of contracted clones (JK15 and JK18) and nonrepetitive DNA (miscellaneous), reactions included 1X Green GoTaq reaction buffer (M7911, Promega), 0.16 mM dNTP mix, 0.8 μM of each primer, 0.5 units of Taq DNA polymerase, and 1.5 μL of the DNA supernatant in a 12.5-μL total reaction volume. PCR products were cleaned using column-based purification prior to Sanger sequencing.

### Spot assays to characterize yeast growth phenotype

Cells were inoculated from fresh YPEG patches into 5 mL of YPD liquid and grown at 30°C overnight. The culture was then reset to an OD_600_ of 0.5, from which five 5-fold serial dilutions were made (i.e. 5^−5^ dilution). From these dilutions, 3 μL was plated on rich media (YPD) plates and 5 μL on selective [5-fluoroorotic acid (5-FOA) and SC-URA] plates. The plates were then incubated at 30°C for 72 h, and images were taken at 48 and 72 h.

### RNA isolation and RT-qPCR analysis

RNA was isolated from log phase cells grown in YPD liquid at 30°C using the YeaStar RNA Kit (Zymo Research R1002) according to manufacturer protocol. Genomic DNA was digested using the TURBO DNA-free kit (Invitrogen AM1907). Complementary DNA (cDNA) was synthesized using 0.5-μg total RNA as template, ProtoScript II Reverse Transcriptase (NEB M0368), and equal amounts of random hexamer primers (Thermo Fisher SO142) and oligo(dT) primers (Thermo Fisher SO131). The cDNA abundance of each strain was quantified using SYBR Green-based qPCR (Thermo Fisher PowerUp A25742) and primers to amplify spliced and unspliced *URA3* transcripts, previously described in Shishkin *et al*. (2009). The delta–delta CT method of relative quantification was used to calculate the relative expression of each region (primer set) compared to the *ACT1* gene as an endogenous control. The relative expression in the long intron strain was set to 1 for comparison. At least 3 biological replicates of cDNA for each strain were used for the analysis. The mean relative expression values and SE are plotted.

### Yeast strain construction for analysis of DNA fragility

Strains to analyze chromosome arm loss were constructed as described (Kim *et al*. 2008). A DNA fragment containing (CCTG/CAGG)_100_ was generated by PCR using p18 plasmid (described above) as template DNA and primer sets with 5′ homology to *LYS2*. JK510/511 and JK512/513 primer sets would result in opposite repeat orientations with respect to *LYS2* (Supplementary Table 4). PCR products were purified by gel extraction and transformed independently into KT119 and KT120 (*MATa*, *his7-2*, *leu2-3,112*, *trp1-Δ*, *ura3-Δ*, *lys2-Δ*, *ade2-Δ*,*bar1-Δ*, *sfa1-Δ*, *cup1-1-Δ*, *yhr054c-Δ*, *cup1-2-Δ*, *lys2::kanMX-URA3*, *ADE2*, *CUP1*, and *SFA1*), as they contained reporter genes relevant to the GCR assay (*CAN1* at the endogenous locus on ChrV and *ADE2* relocated to ChrV as in Fig. 4) as well as selectable markers for transformant screening (*URA3* and *KanMX*). Replacement of the “core” sequence disrupting *LYS2* would result in 5-FOA^R^ and G418^S^ yeast cells. In addition, we cotransformed the starting strains with a CRISPR plasmid that would direct a DSB at *KanMX* to increase the likelihood of successful targeting. These transformant clones were analyzed for repeat length by PCR using primers JK514 and JK515 (PCR conditions below) and Sanger sequencing (Genewiz). YJK302-3 is a spontaneous expansion that was verified by sequencing to be (CAGG)_138_.

For PCR analysis of CCTG/CAGG repeat length in GCR strains using primers JK514 and JK515 (655-bp product for (CCTG)_100_), reactions included 1× Green GoTaq reaction buffer (M7911, Promega), 0.2 mM dNTP mix, 0.8 μM of each primer, 3% DMSO, 0.75 units of Taq (Thermo Scientific EP0402), 0.3 units of Phusion Hot Start II DNA polymerase (F549, Thermo Fisher Scientific), and 1.5 μL of the DNA supernatant in a 20-μL total reaction volume. Thermocycling conditions were 60 s at 95°C, followed by 32 cycles of 30 s at 95°C, 20 s at 60°C, and 60 s at 72°C, concluding with 10 min at 72°C.

### Measurement of chromosome arm loss rates

Rates of chromosome arm loss were determined through fluctuation assays. HMK1/2 strains containing (GAA)_5_ repeat tracts served as a negative, nonfragile control. Verified strains were initially grown on YPEG to select against petite mutants. Single colonies were grown on rich media (YPD) plates supplemented with 50-μg/mL uracil (referred to as DU) for 72 h at 30°C. Whole, individual colonies were suspended in 200-μL sterile water. To select for Can^R^Ade− clones, 100 μL of the cell suspension was plated on synthetic media with low adenine (5 μg/mL) and 60-μg/mL canavanine (Sigma C9758) with 2% glucose, 0.67% yeast nitrogen base, and 2% agar. The cell suspension was serially diluted to plate on YPD media (100 μL of 10^−5^ dilution) for determination of total cell number. For each independent experiment, at least 12 independent colonies were analyzed. Colonies growing on DU that had an initial contraction or expansion, assessed via repeat length PCR, were excluded from the analysis.

Cells were incubated at 30°C and grown for 7 days. Colony counts occurred on days 3, 5, and/or 7. The same trends were observed on each day, so the data for day 3 colony counts were used to determine rates. Rates and 95% CIs were calculated using the MSS-MLE method as described above for large-scale contractions. Because of the larger 95% CIs associated with these arm loss rates (∼10^−8^), the average of 3 independent experiments was used to calculate a mean arm loss rate and SE. An unpaired *t*-test was used to determine statistical significance compared to the control (GAA)_5_ strain (HMK1/2).

The same method was used to determine *CAN1* mutation rate except that the number of canavanine resistant, white clones (Ade+) was used for fluctuation analysis.

## Results

### A system to investigate CAGG/CCTG repeat contractions

**Fig. 1.**
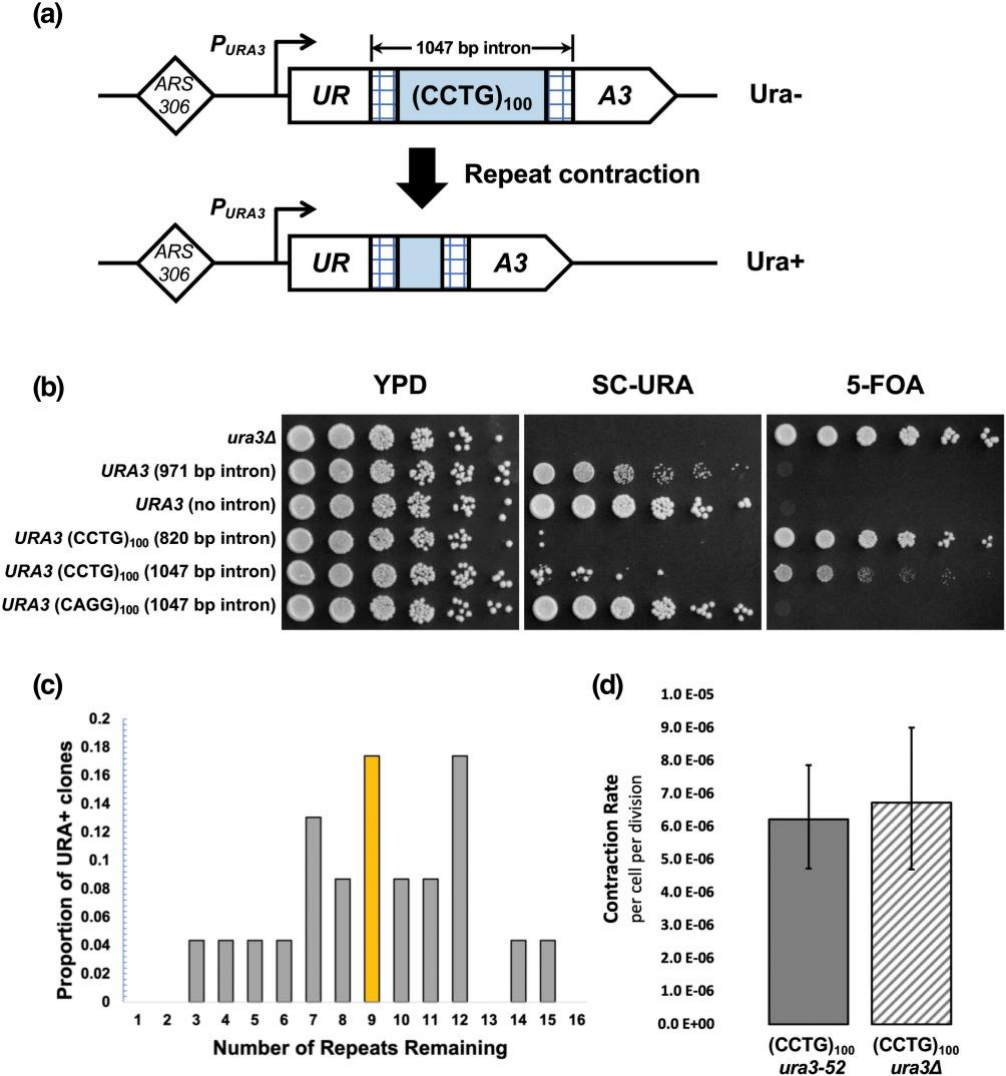
Experimental system to study large-scale CCTG repeat contractions in vivo. a) DNA repeats are cloned into the artificial intron, derived from *ACT1*, of a *URA3* reporter gene. The reporter gene is integrated ∼1-kb downstream of the replication origin *ARS306*. The starting strain with (CCTG)_100_ is Ura−. Repeat contraction renders the cells Ura+. b) Spot assays to evaluate the growth phenotypes of yeast strains with (CCTG)_100_ and (CAGG)_100_ repeats as well as nonrepetitive DNA. c) Distribution of remaining repeat length in Ura+ clones, evaluated by PCR and Sanger sequencing. The median number of repeats was 9. d) Rate of large-scale contraction for (CCTG)_100_ strains, shown with 95% CIs. Rate is calculated using the number of Ura+ clones beginning with 12 independent cultures and the MSS-MLE with a correction for sampling and plating efficiency.

To investigate CAGG/CCTG repeat instability, we employed a system previously used to study large-scale GAA repeat expansions (Shishkin *et al*. 2009) and contractions (Khristich *et al*. 2020), which are responsible for Friedreich's ataxia (FA). The yeast system mimics the intron location of GAA repeats in the gene responsible for FA, wherein *URA3* under the control of its native promoter is artificially split by an intron containing (GAA)_100_ repeats and used for forward selection. Large-scale GAA expansions increase the intron length beyond the yeast splicing threshold of ∼1 kb (Yu and Gabriel 1999), which impairs *URA3* splicing and renders cells resistant to the drug 5-fluoroorotic acid (5-FOA). Cells can also become 5-FOA^R^ through spontaneous point mutations, insertions and deletions, or other mechanisms that inactivate *URA3* expression. We adapted the system by cloning (CCTG)_100_ repeats into the artificial intron of *URA3* (Fig. 1a). The cassette was integrated into chromosome III, ∼1 kb away from *ARS306*, a replication origin that is efficient and activated early in the S phase. In these cells, CCTG is on the sense strand, as it is for *CNBP*, and lagging strand template for DNA replication. We also constructed an equivalent yeast strain with (CAGG)_100_ on the lagging strand template to investigate the effect of repeat orientation.

Unexpectedly, we found that the 2 orientations led to distinct growth phenotypes with respect to uracil auxotrophy (Fig. 1b), despite the total intron length of both strains being identical (1,047 bp). The (CAGG)_100_ orientation resulted in a Ura+ phenotype equivalent to *URA3* with no intron: growth occurred on synthetic media lacking uracil, whereas no growth occurred on 5-FOA media. In contrast, the (CCTG)_100_ orientation showed a predominantly Ura− phenotype with some distinct Ura+ papillae. To exclude the possibility that the (CCTG)_100_ strain was Ura− because the intron length exceeded the yeast splicing threshold, we generated yeast with (CCTG)_100_ but a shorter overall intron length (820 bp). The growth phenotype was still Ura− in this modified strain (Fig. 1b), though there was greater resistance to 5-FOA than for the longer intron strain. Since the Ura− phenotype was dependent on the presence of CCTG repeats rather than exclusively intron length, we used the (CCTG)_100_ strain with the 1,047-bp intron length for subsequent analyses of the CCTG orientation to maintain direct comparison to the (CAGG)_100_ strain.

**Fig. 2.**
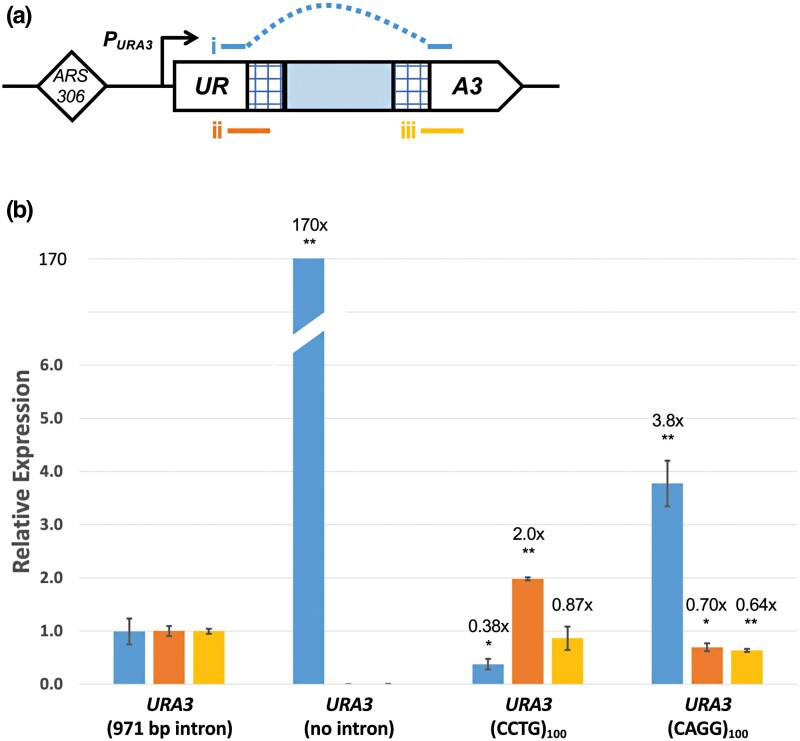
Comparison of *URA3* reporter gene expression in strains with and without CAGG/CCTG repeats. a) Representation of PCR amplicons to evaluate: i) spliced *URA3*, ii) 5′ unspliced *URA3*, and iii) 3′ unspliced *URA3*. Dashed line denotes that primers will not anneal to the intron sequence. b) Relative expression of spliced and unspliced *URA3* transcripts compared to *ACT1* endogenous control in 4 yeast strains. Bar graph colors correspond to amplicon colors as in a). The relative expression in the long intron strain was set to 1 for comparison, and the numerical fold difference is in comparison to the long intron strain. At least 3 biological replicates of cDNA for each strain were used for the analysis. The mean relative expression values and SE are plotted. Statistical significance was evaluated by unpaired *t*-test (*P < 0.05; **P < 0.01) for each primer set in the no intron, (CCTG)_100_, or (CAGG)_100_ cDNA sample compared to the long intron strain.

We hypothesized that the (CCTG)_100_ orientation inhibited functional *URA3* expression and that Ura+ clones in the spot assay were due to spontaneous repeat contractions that restored its expression. We performed RT-qPCR to evaluate how transcript levels corresponded to uracil auxotrophy. We observed that a strain with no intron showed >100-fold increase in *URA3* transcript abundance compared to the no-repeat intron (971 bp) and (CCTG)_100_ strains (Fig. 2). The (CCTG)_100_ strain showed a decrease (2.6-fold) in spliced *URA3* transcript compared to the no-repeat intron strain, providing evidence that a splicing defect in the CCTG orientation contributes to its Ura− phenotype. In contrast, the (CAGG)_100_ strain showed a 3.8-fold increase in spliced *URA3* transcript compared to the no-repeat intron strain. This difference in *URA3* expression between the 2 orientations was unexpected and is further described in the Discussion.

The difference in gene expression between CAGG and CCTG orientations also restricted what type of instability events, in theory, could be analyzed through selection. For example, since the (CAGG)_100_ strain is Ura+ and 5-FOA^S^, we hypothesized that some 5-FOA^R^ clones might arise from large-scale CCTG expansions that cause the intron length to exceed the splicing threshold. We determined the rate of 5-FOA resistance (per cell per division) for the (CAGG)_100_ strain to be 5 × 10^−7^ (Supplementary Fig. 2). However, upon PCR analysis of 5-FOA^R^ clones, fewer than 10% showed larger PCR products (Supplementary Fig. 2), indicating that the system needs to be fine-tuned to investigate (CAGG)_100_ large-scale expansions in a robust manner. Thus, we focused on the (CCTG)_100_ strain and whether cells that changed from Ura− to Ura+ could be evaluated to study large-scale contractions.

To measure the rate of Ura+ clone formation, we conducted fluctuation tests on the (CCTG)_100_ orientation strain. After performing PCR analysis on 24 Ura+ clones using primers flanking the repeats and sequencing the PCR products, we found that the remaining number of repeats ranged from 3 to 15, with a median of 9 repeats (Fig. 1c). This indicates a massive net contraction of over 80 repeats. We calculated the rate of Ura+ clone formation to be 6 × 10^−6^ per replication (Fig. 1d), which we designate as the WT rate of large-scale contraction based on the PCR and sequencing analyses. To confirm that Ura+ clones were not due to gene conversion events with the endogenous *ura3–52* allele on chromosome V, which contains a Ty1 retrotransposon insertion, we integrated the (CCTG)_100_ cassette into an isogenic background with a full *URA3* deletion. We observed an equivalent rate of Ura+ clone formation (Fig. 1d), indicating that they are primarily (if not exclusively) due to large-scale contractions. Next, we focused on delineating the genetic control of large-scale (CCTG)_100_ contractions.

### Impairment of lagging strand synthesis does not affect large-scale CCTG repeat contractions

**Fig. 3.**
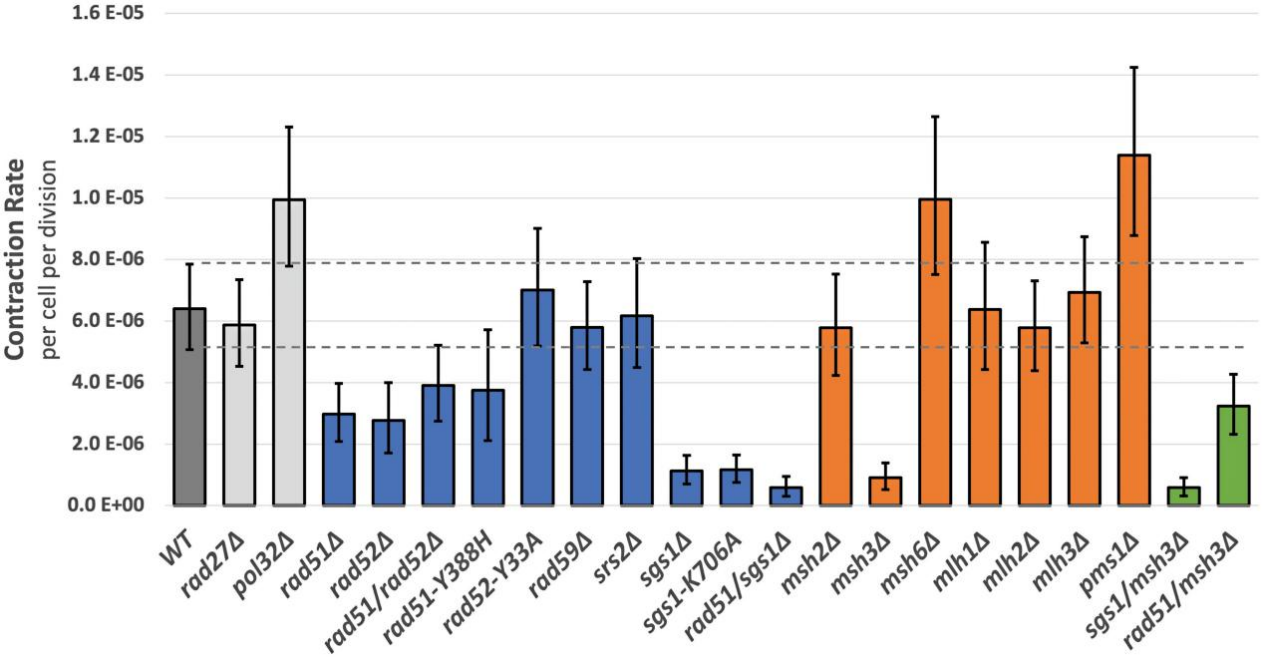
Genetic analysis of large-scale CCTG repeat contractions. Rate of large-scale contraction for (CCTG)_100_ strains, shown with 95% CIs (dashed lines for WT strain). Rate is calculated using the number of Ura+ clones beginning with 12 independent cultures and the MSS-MLE with a correction for sampling and plating efficiency.

The previous study investigating large-scale contractions of GAA repeats found that contractions occur during DNA replication rather than DNA repair pathways (Khristich *et al*. 2020). The median contraction size was ∼60 repeat units, and a mechanism involving the bypass of a transient triplex DNA structure during lagging strand synthesis was proposed. Notably, there was a >40-fold increase in large-scale GAA contractions following the loss of Rad27, a 5′ to 3′ exonuclease and 5′ flap endonuclease that processes single-stranded flaps during Okazaki fragment maturation. In contrast, we found that large-scale CCTG contraction rate in the *rad27Δ* strain was indistinguishable from WT (Fig. 3; Supplementary Table 5). Furthermore, we found only a small increase (1.6-fold) by knocking out the processivity unit of DNA polymerase δ (*pol32Δ*). Because these key proteins in DNA replication and lagging strand DNA synthesis showed no effect on large-scale CCTG contractions, we focused our subsequent genetic analysis on DNA repair pathways.

### HR is involved in large-scale CCTG repeat contractions

Various DNA repeats have been shown to elevate HR in plasmid-based systems, resulting in expansions and contractions. For example, using an *E. coli* intramolecular plasmid system, CAGG/CCTG repeats increased recombination crossover frequencies with expansions being more prevalent than contractions (Dere and Wells 2006). Similar results were observed for CAG/CTG (Napierala *et al*. 2002) and GAA/TTC (Napierala *et al*. 2004) repeats using this *E. coli* system, though contractions were more prevalent. The role of HR on chromosomal DNA repeats is more varied depending on tract length and microsatellite sequence. Loss of Rad51 recombinase and Rad52, which facilitates Rad51 loading, had no effect on short tracts of (CAG)_13_ or (CAG)_25_ repeats (Miret *et al*. 1998; Bhattacharyya and Lahue 2004) whereas it did enhance small-scale expansions (Sundararajan *et al*. 2010) and contractions (Su *et al*. 2015) of (CAG)_70_, indicating a protective effect of HR. In contrast, large-scale expansions of (CAG)_140_ were dependent on HR through a proposed break-induced replication mechanism (Kim *et al*. 2017). However, the loss of Rad52 had no effect on large-scale GAA repeat expansions (Shishkin *et al*. 2009) and a mildly protective effect (2-fold increase in *rad52Δ*) on large-scale GAA contractions (Khristich *et al*. 2020).

We observed a 2.2-fold decrease in large-scale contraction rates in *rad51Δ* and a 2.3-fold decrease in *rad52Δ* compared to WT (Fig. 3), demonstrating that HR promotes this process. We found that this decrease was consistent using 2 analytical methods: nonoverlapping 95% CIs and comparison of multiple trials encompassing 59 independent cultures (Supplementary Fig. 3). Furthermore, we tested whether Rad51 and Rad52 might have a compensatory effect or overlapping functions. The *rad51Δ rad52Δ* double mutant showed a comparable, epistatic (and not synergistic) 1.6-fold decrease to the single mutants, indicating that their roles are in the same genetic pathway. We tested specific mutants to investigate the roles of these proteins further. Rad51-Y388H is defective in interactions with Rad52 as well as Srs2 helicase (Seong *et al*. 2009). This mutant demonstrated a 1.7-fold decrease in large-scale contraction rate. Rad52-Y33A is defective in DSB repair, as demonstrated by sensitivity to γ-irradiation, but proficient in spontaneous mitotic recombination such as heteroallelic recombination. The large-scale contraction rate of Rad52-Y33A was indistinguishable from WT. We further evaluated the role of the Rad52 homolog Rad59, which is required for single-strand annealing, and found no difference in large-scale contraction rate compared to WT.

### Srs2 helicase is not required for large-scale CCTG repeat contractions in unchallenged cells

Srs2 is a DNA helicase that functions in many aspects of DNA replication, repair, and recombination. Notably for DNA repeat instability, Srs2 can unwind CAG, CTG, and CGG substrates in vitro (Bhattacharyya and Lahue 2005). 2D gel-electrophoretic analysis of replication intermediates demonstrated that Srs2 is required in vivo for replication fork progression through these TNRs (Kerrest *et al*. 2009). Loss of Srs2 function caused an increase in small-scale expansions of (CAG)_25_, (CTG)_25_, and (CGG)_25_ repeats (Bhattacharyya and Lahue 2004) and nonselective expansions and contractions of (CTG)_55_ (Kerrest *et al*. 2009), though it had no effect on large-scale expansions of (CAG)_140_ (Kim *et al*. 2017). Because CCTG/CAGG repeats can also form hairpin structures, we investigated loss of Srs2 function in our assay, predicting that there would be an increase in large-scale contractions if Srs2 unwound CCTG/CAGG repeats due to replication bypass of structures formed on the template strand. We found that the rate of large-scale CCTG contractions in *srs2Δ* did not differ from WT (Fig. 3).

Because Srs2 has an anti-recombinase role and displaces Rad51 filaments during HR, we tested whether loss of Srs2 function would influence large-scale CCTG contractions when cells were challenged with replication stress, specifically DSBs. When *srs2Δ* cells were treated with camptothecin (CPT), a topoisomerase I inhibitor that results in breaks during DNA replication, rates of large-scale CCTG contractions increased over a range of concentrations (Supplementary Fig. 4; Supplementary Table 6). For example, comparing *srs2Δ* to WT, there was a 4.8-fold increase at 10 μM CPT and a 5.7-fold increase at 50 μM CPT. In addition, 50 μM CPT increased the contraction rate of *srs2Δ* cells 11.8-fold compared to untreated *srs2Δ* cells, revealing a dose-dependent effect of DNA damage on the role of Srs2 function in large-scale CCTG contractions. Furthermore, we observed a similar increase in contraction rates with hydroxyurea (HU) treatment (Supplementary Fig. 4; Supplementary Table 6), which causes replication fork stalling and uncoupling of the replication fork polymerases and helicase that can result in DSBs. At 50 mM HU, *srs2Δ* showed a 3.1-fold increase compared to WT. For both CPT and HU treatments, there was also a ∼2-fold increase in contraction rate at all doses in the WT strain compared to no treatment. We propose that the increase is indicative of DSBs that are instigated during the S phase and aberrantly repaired to generate large repeat contractions (see Discussion).

### Sgs1 helicase is required for large-scale CCTG repeat contractions

The RecQ helicase, Sgs1, plays various roles in DNA repair and recombination in budding yeast (Gupta and Schmidt 2020). During recombination, Sgs1 plays an early role in long-range resection at a DNA DSB and was shown to be required for heteroduplex rejection during single-strand annealing (Sugawara *et al*. 2004). Through biochemical assays, purified Sgs1 was demonstrated to unwind a broad range of DNA structures including Holliday junctions (Cejka and Kowalczykowski 2010) and G-quadruplexes (Huber *et al*. 2002). Long (CTG)_75_ repeats were shown to be stabilized by Sgs1, as loss of function led to increased expansions and contractions under nonselective conditions (Kerrest *et al*. 2009).

The absence of Sgs1 had a considerable effect on large-scale CCTG contraction rate, as *sgs1Δ* showed a 5.7-fold decrease compared to WT (Fig. 3). Interestingly, Sgs1 is required for large-scale contractions even under unperturbed conditions, which is opposite the effect displayed by Srs2 when treated with replicative stressors. To better understand the role of Sgs1 in repeat contractions, we constructed and analyzed a helicase-defective mutation Sgs1-K706A. This strain also displayed a 5.5-fold decrease in contraction rate compared to WT (Fig. 3), indicating that Sgs1 helicase activity is required for its effect on large-scale CCTG contractions. We evaluated the contraction rate in the *rad51Δ sgs1Δ* double mutant and found a 10.8-fold decrease in contraction rate compared to WT (Fig. 3). This was a slight decrease compared to the *sgs1Δ* mutant, though the 95% CIs are overlapping.

Having established a requirement for Sgs1 on large-scale CCTG repeat contractions, we investigated whether its role was through facilitating 5′ DNA end resection, a key processing step to promote DSB repair by HR. The Mre11-Rad50-Xrs2 (MRX) complex localizes to a DSB, upon which the Mre11 nuclease can initiate resection. Sae2 has also been shown to be involved in short-range resection, though both Mre11 and Sae2 have been shown to be dispensable for the resection of DNA ends overall. Long-range, more processive resection is mediated by Sgs1-Dna2 or Exo1 pathways, which may have redundant roles. We found that single mutants of *mre11Δ*, *sae2Δ*, and *exo1Δ* each displayed large-scale contraction rates that were not significantly different from WT (Supplementary Fig. 5). Sgs1 interacts with Top3 and Rmi1 to form the STR complex, which enhances DNA end resection and resolves recombination intermediates. We did not observe an effect on large-scale contraction rates in the *rmi1Δ* mutant.

Because the nuclease domain of Dna2 is essential, its role in DNA resection could not be evaluated through direct knockout. We tested the *dna2-H547A* mutant, which is described as nuclease-attenuated, though it maintains ATPase and helicase activity (Lee *et al*. 2000). We found that the mutant displayed a 12-fold elevated rate, suggesting that Dna2 nuclease activity plays a protective role in CCTG repeat contractions (Supplementary Fig. 5).

### MMR proteins are differentially involved in large-scale CCTG repeat contractions

DNA mismatch repair (MMR) is initiated by the MutSα and MutSβ complexes, formed by the Msh2-Msh6 and Msh2-Msh3 heterodimers, respectively. MutSα recognizes single-base mismatches, while MutSβ recognizes insertion/deletion loops. MMR proteins have been implicated in CAG/CTG repeat instability. Specifically, CAG/CTG TNRs were stabilized in the *msh3Δ* mutant (Williams and Surtees 2015). Loss of Msh2 and Msh3, though not Msh6, was shown to decrease expansions in mouse models (Manley *et al*. 1999; van den Broek *et al*. 2002; Foiry *et al*. 2006). These results were corroborated in a human cell culture system (Gannon *et al*. 2012). However, the loss of MMR proteins had no effect on large-scale (>60 repeats) expansions of CAG/CTG repeat in a yeast experimental system (Kim *et al*. 2017).

In evaluating large-scale CCTG contractions, we found that loss of MMR proteins demonstrated differential effects (Fig. 3). The strongest effect was displayed by *msh3Δ,* which showed a 7.0-fold decrease in contraction rate. In contrast, *msh2Δ* showed no difference and *msh6Δ* a slight increase (1.6-fold) in contraction rate, both of which were not significant. Following mismatch recognition, proteins in the MutL family function as heterodimers to catalyze repair. They also have roles outside of MMR such as in meiotic recombination (Pannafino and Alani 2021). Large-scale contraction rates in *mlh1Δ*, *mlh2Δ*, *mlh3Δ*, and *pms1Δ* single mutants were not significantly different from WT.

To evaluate the relationship of Msh3 with Sgs1 and Rad51, we constructed double mutants and tested their effect on large-scale CCTG repeat contractions. The *sgs1Δ msh3Δ* mutant showed an 11-fold decrease in contraction rate compared to WT, a slight decrease compared to the single mutants though the 95% CIs overlap. In contrast, the *rad51Δ msh3Δ* mutant showed a rate (2.0-fold decrease) comparable to the *rad51Δ* single mutant, evidence of an epistatic relationship where Rad51 functions upstream of Msh3 in the genetic pathway. We synthesize these genetic results in the Discussion.

### CCTG repeats increase chromosomal fragility in an orientation-dependent manner

**Fig. 4.**
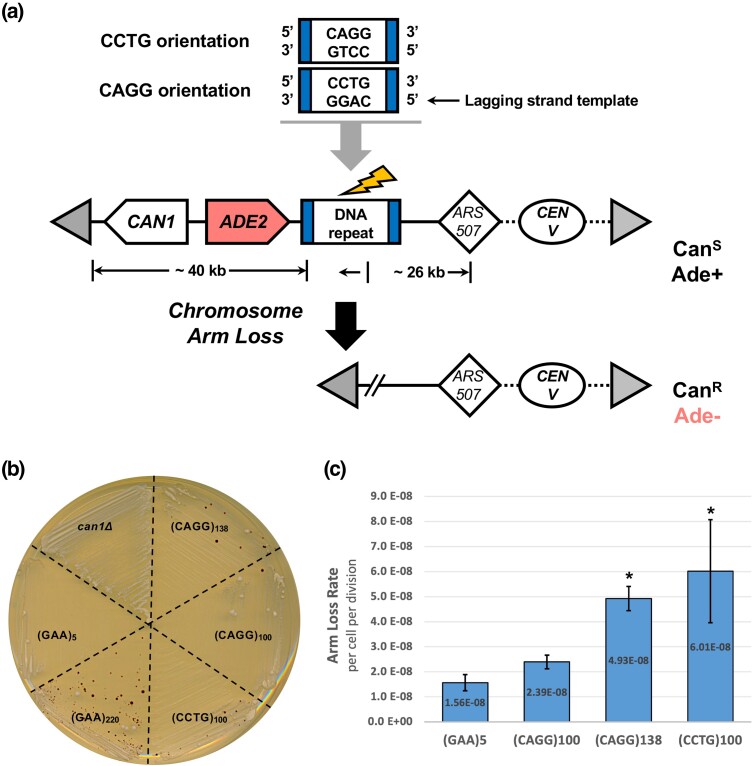
CCTG repeats elevate chromosomal fragility in an orientation-dependent manner. a) Experimental system to study repeat-induced DNA fragility via chromosomal arm loss. CCTG and CAGG repeats were integrated at the reporter locus, *lys2* (blue). DSB formation followed by telomere addition (gray triangle) will result in increased arm loss frequency of the left chromosomal arm. This region (∼40 kb) contains no essential genes and the selectable markers *CAN1* and *ADE2*. Mutations in these genes result in canavanine-resistant (Can^R^) and adenine auxotroph clones (Ade−) that appear red when plated on selective media. The closest origin of replication is *ARS507*. As such, the lagging strand template for DNA replication is noted at the top of the figure. The CCTG and CAGG orientations refer to their placement on the lagging strand template. b) Growth of strains on media containing canavanine and low adenine (5 μg/mL). (GAA)_5_ and (GAA)_220_ strains are described in (*41*). c) CCTG/CAGG repeats elevate arm loss rates. Arm loss rates of (GAA)_5_, (CAGG)_100_, (CAGG)_138_, and (CCTG)100 from 3-day incubation on selective media. Arm loss rates were calculated with FluCalc, which uses the MSS-MLE model with a correction for sampling and plating efficiency. Error bars indicate SE from 3 independent experiments. Statistical significance was evaluated by unpaired *t*-test (*P < 0.05) for each CAGG/CCTG strain compared to the nonfragile (GAA)_5_ control.

To confirm DSB formation caused by CAGG/CCTG repeats, we used a genetic assay (Kim *et al*. 2008) to determine the rates of chromosomal fragility in both repeat orientations. This assay works by integrating DNA repeats into a relocated *lys2* locus on ChrV (Fig. 4a), where DNA breakage can lead to a telomere-proximal deletion (arm loss). This region does not contain essential genes but does contain *CAN1* and *ADE2*. This allows for the selection of canavanine-resistant (Can^R^) and Ade− clones, which appear red, and permits subsequent calculation of DNA fragility rates. For this arm loss assay, we designate repeat orientation as previously described and name the repeat sequence that is on the lagging strand template for DNA replication. This orientation is determined by *ARS507*, which was characterized by 2D gel analysis of replication intermediates (Zhang *et al*. 2013).

We tested DNA fragility in (CCTG)_100_ and (CAGG)_100_ strains as well as a (CAGG)_138_ strain that was isolated as a spontaneous expansion during strain construction. We observed an increase in Can^R^Ade− clones that was above the background levels of a (GAA)_5_ negative control strain but noticeably less than (GAA)_220_ (Fig. 4a). Through fluctuation analyses, we quantified arm loss rates of the (CCTG)_100_ and (CAGG)_138_ orientations to be significantly increased compared to the (GAA)_5_ negative control (*P* < 0.05) whereas (CAGG)_100_ showed an upward trend that was not significantly different (Fig. 4c). The rates of *CAN1* mutation alone among the CAGG/CCTG strains were not significantly different compared to the (GAA)_5_ negative control (Supplementary Fig. 6).

## Discussion

We have established an experimental system to characterize massive contractions of CCTG repeats, showing by genetic analysis that large-scale CCTG contractions occur via HR. Through analysis of double mutants, we characterize epistatic relationships between genes involved in large-scale CCTG contractions. Furthermore, we provide the first evidence that CAGG/CCTG repeats increase DSB formation and chromosomal fragility in vivo.

Large-scale CCTG contractions were not dependent on proteins involved in lagging strand synthesis (Pol32) and Ozakaki fragment processing (Rad27). This demonstrates that the genetic control is distinct from large-scale GAA repeat contractions, which were substantially increased in *pol32Δ* and *rad27Δ* mutants. However, we did observe an increase in CCTG contractions in the *dna2-H547A* mutant, which may involve Okazaki fragment processing by cleaving long 5′ flaps or another role in DSB end processing (discussed below). GAA repeats form a triplex structure consisting of a GAA/TTC double helix with standard Watson Crick base pairing and, in its most stable configuration, a third GAA homopurine strand that anneals via reverse Hoogsteen hydrogen bonds. Large contractions of GAA repeats were proposed through DNA polymerase occasionally bypassing this secondary structure (Khristich *et al*. 2020). However, CAGG/CCTG repeats are not predicted to form triplex structures. Rather, there is gel electrophoretic evidence for hairpins (more stable in the CAGG orientation) (Dere *et al*. 2004) and slipped strand DNA (Edwards, Sirito, *et al*. 2009). High-resolution NMR studies with nucleotide substitution experiments have illuminated additional secondary structures such as dumbbells, minidumbbells, and loops, which may interchange configurations rapidly. Though strand slippage on the template strand is likely to contribute to some repeat contractions, these are likely to be 1-to-3 unit deletion events.

**Fig. 5.**
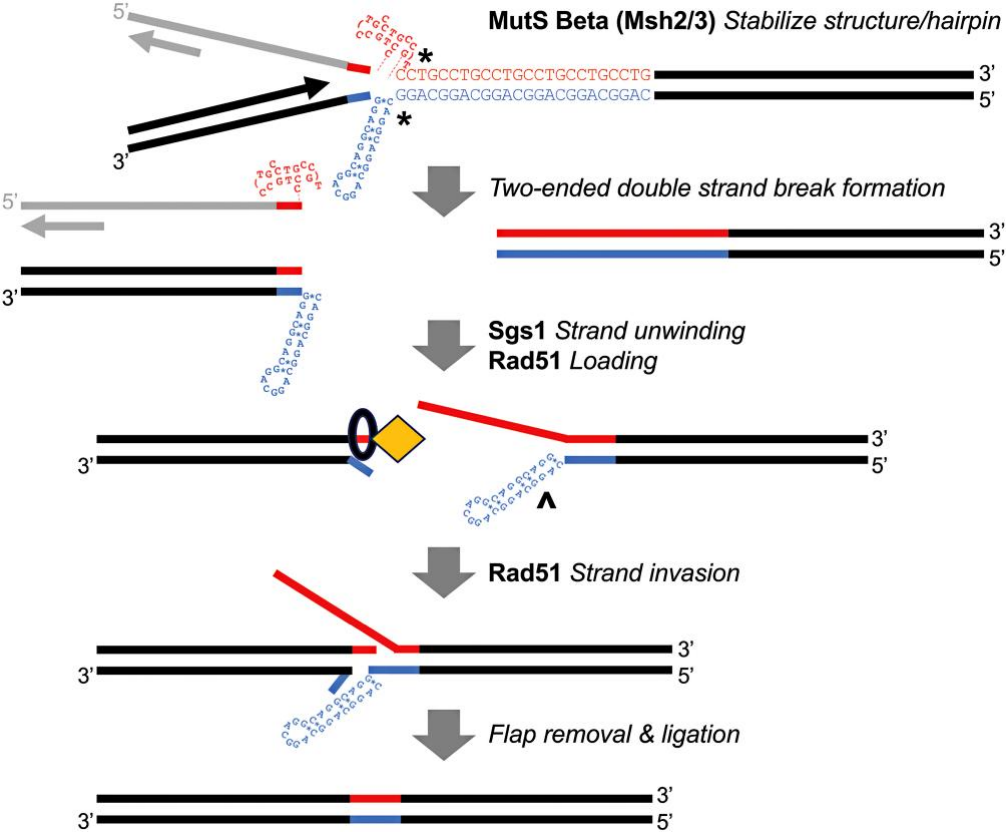
Proposed model of CCTG repeat contraction in budding yeast. During DNA replication, secondary structures such as dumbbells and hairpins form on ssDNA. ssDNA formation could be elevated due to the uncoupling of DNA polymerases and helicase at the replication fork when replicating through the DNA repeats (red/blue tracts). DNA DSBs occur at the CAGG/CCTG repeats, possibly mediated by a structure-specific nuclease(s). Sgs1 helicase promotes DNA strand unwinding, translocating in a 3′ to 5′ direction. At the DSB end close to the start of the repeat tract, the 5′ end will be displaced by Sgs1 (hairpin omitted for clarity). At the 3′ end, Rad52 (black circle) will help load Rad51 (yellow diamond) in a 5′ to 3′ direction. At the other DSB end in the absence of Dna2 endonuclease activity, Sgs1 can unwind the repeat DNA more extensively. Hairpin formation can occur on the newly displaced CAGG strand (^). Rad51 and Rad52 promote DNA invasion of the CCTG 3′ overhang to the homologous template. Because of the repetitive nature of the DNA template, out-of-register alignment toward the distal end of the repeat tract will result in a massive contraction. Importantly, only events where the invading DNA end aligns toward the edge of the repeat tract on the template strand will result in a large enough repeat contraction for Ura+ selection. MutSβ may be involved in stabilizing secondary structures and/or processing flap removal following strand annealing.

Since we observe very large CCTG repeat contractions exceeding 80 repeat units, we describe a model for repeat contractions that is distinct from replication slippage (Fig. 5). Both CPT and HU treatments elevate contraction rates even in the WT strain. Thus, we propose that DSBs are generated during the S phase, though indirect effects of the replication stress response are a possibility that remains to be tested. Because of the high GC content and structure-forming potential of the repeats, replication through the repeat tract may result in the uncoupling of the replication fork helicase and polymerase. This uncoupling could increase the persistence of single-stranded DNA (ssDNA) and exacerbate secondary structure formation, structures that could be stabilized by MutSβ (Tian *et al*. 2009; Zhao *et al*. 2015). A 2-ended DSB could then be generated by structure-specific nucleases acting at the distortion(s) in B-DNA, though the nucleases involved remain to be identified. Next, the DSB is repaired by HR, analogous to a fork restart mechanism. Because of the repetitive nature of the DNA template, out-of-register alignment will result in a massive contraction. Importantly, only events where the invading DNA end aligns toward the edge of the repeat tract on the template strand will result in a large enough repeat contraction for Ura+ selection. We believe such events are more likely due to the hairpin-forming potential in the CAGG orientation. Next, we summarize some of our genetic analysis to describe how Rad51, Rad52, Sgs1, Dna2, and Msh3 may be acting.

Large-scale CCTG contractions were dependent on Rad51 and Rad52, though the effect was modest (a 2-fold decrease in knockouts). In a previous study examining single-strand annealing within the ribosomal DNA array, *RAD52* was not essential, and it was proposed that the presence of large amounts of homology enabled Rad52-independent DSB repair (Ozenberger and Roeder 1991). The simple tandem repeat nature of the CCTG locus may make Rad51 and Rad52 dispensable for homology search leading to DSB repair. In our double mutant analysis, none of the effects were synergistic. Some double mutants were clearly epistatic (*rad51Δ rad52Δ* and *rad51Δ msh3Δ*), and others were epistatic or possibly additive (*rad51Δ sgs1Δ* and *sgs1Δ msh3Δ*). To harmonize these results, we interpret a genetic relationship with Sgs1 upstream of Rad51.

Among its various roles in DNA repair, Sgs1 is known to promote 5′ DNA resection to form a longer 3′ ssDNA tail via its 3′ to 5′ helicase/translocation activity (Cejka and Kowalczykowski 2010). Sgs1 is also proposed to help translocation of the invading strand along a Holliday junction through interaction with Rad51, perhaps facilitating the search for homology. Because homology search at the CCTG repeats may not rely solely on Rad51 and Rad52, as described above, we investigated whether genes involved in DNA resection would also be required for large-scale CCTG contractions. We did not find an effect by deleting *MRE11* (MRX complex), *SAE2*, *RMI1* (STR complex), or *EXO1*. Thus, there may be redundant exonucleases that, along with Sgs1, generate the 3′ overhangs for HR. We tested the *dna2-H547A* mutant that has been shown to have diminished endonuclease activity but unaffected ATPase and helicase activity (Lee *et al*. 2000). We found that contractions were increased 7.5-fold in the *dna2-H547A* mutant, which was the opposite effect in the *sgs1Δ* mutant. The role of *DNA2*, which is essential, remains to be elucidated further. However, because Sgs1 and Dna2 physically interact (Cejka *et al*. 2010), one possibility is that Dna2 counteracts Sgs1 activity in promoting CCTG contractions. Thus, in the absence of Dna2 endonuclease activity, Sgs1 can unwind the repeat tract more extensively, and subsequent hairpin formation on the newly unwound CAGG strand would then increase the likelihood of strand invasion at the distal edge of the repeat tract (Fig. 5). In contrast, when Dna2 activity is unperturbed, DNA unwinding and strand resection may be more limited since Rad52 would be loaded in a 5′ to 3′ direction from the newly resected end. In this scenario, a single-strand annealing mechanism may contribute to small-scale contractions that are not detected in our selectable system (Supplementary Fig. 7). These models remain to be tested with further single and double mutant analysis.

MMR genes have been widely studied to investigate microsatellite DNA repeat instability, and their described roles depend on various factors including repeat sequence, length, and scale of expansion (Sia *et al*. 1997). We found that Msh3 is necessary for large-scale CCTG contractions, whereas Msh2 had no effect and Msh6 showed a slightly protective effect. As Msh2/3 forms a heterodimer, it is unclear why the *msh2Δ* mutant did not show a concomitant effect as *msh3Δ*. One possibility is that the Msh2/6 heterodimer plays an opposing role, which was proposed in a previous study investigating CAG/CTG repeat expansions. However, in that study, the absence of a phenotype in *msh2Δ* mutants was explained by opposing effects of Msh3 and Msh6 on lagging strand synthesis (Kantartzis *et al*. 2012), a pathway we did not observe to play a major role in large-scale CCTG contractions. Msh2/3 has been shown to bind insertion/deletion mismatches in yeast (Habraken *et al*. 1996). Thus, one possibility is that the heterodimer is involved in recognizing the DNA lesion that contributes to the DNA break. However, our double mutant analysis also places *RAD51* genetically upstream of *MSH3*. Thus, Msh3 may also be involved in a later step such as flap removal, like its described role in single-strand annealing (Sugawara *et al*. 2004; Bhargava *et al*. 2016). Characterizing the role of Msh3 warrants further investigation to clarify the role of MMR on large-scale CCTG contractions.

Although the primary focus of this study was to investigate CCTG DNA repeat instability, our results show an intriguing difference of repeat orientation on gene expression. Namely, the (CAGG)_100_ orientation showed increased spliced *URA3* transcript compared to the (CCTG)_100_ orientation, which corresponded to growth on media lacking uracil. Based on the qPCR analysis, we propose that the CCTG orientation impairs the splicing of the CCUG repeat containing RNA since there is a greater abundance of 5′-unspliced *URA3* transcript in the (CCTG)_100_ orientation. However, other mechanisms remain to be explored such as intron retention, which was demonstrated through transcriptome analysis from DM2 patient samples (Sznajder *et al*. 2018), or RNA cleavage, which was previously shown with (UUC)_n_ RNA in *E. coli* (Krasilnikova *et al*. 2007).

Finally, we have shown that disease-causing lengths of CCTG repeats cause chromosomal fragility in vivo. Because the assay requires DSB formation followed by DNA repair via telomere addition or break-induced replication with a nonhomologous template, the rate of arm loss is likely to be an underestimate of total DSB formation induced by CCTG repeats. Given that CCTG repeat length can reach thousands in DM2 patients, it is possible that DSB repair mechanisms might also contribute to such massive repeat expansions in human cells. Our (CCTG)_100_ strain does not permit the selection of large-scale expansions since the starting strain is already Ura−. Thus, new experimental models will need to be developed and fine-tuned to analyze large-scale CCTG repeat expansions in a systematic way. In addition, it was shown that CCTG/CAGG tracts decreased significantly in the affected offspring of DM2 patients (Day *et al*. 2003), and one intriguing possibility is that such contractions result from recombination following programmed DSBs during meiosis. One bioinformatic study proposed that the DM2 CCTG repeats may have originated from an AluSx element insertion (Kurosaki *et al*. 2012). As long-read sequencing of repetitive DNA continues to advance, it will be important to evaluate the prevalence of CCTG repeats in the human genome and their molecular properties of expansions, contractions, and DNA fragility.

## Supplementary Material

jkad257_Supplementary_Data

## Data Availability

All data, yeast strains, and plasmids underlying this article will be shared on request to the corresponding author. Supplementary Tables 1, 3, and 4 contain a list of primers used for CCTG/CAGG repeat cloning and RNA analysis, genetic analysis and mutant strain construction, and GCR strain construction, respectively. Supplementary Table 2 contains a list of the strains used. Supplemental material available at G3 online.
