# Supplementary material for: Massive contractions of myotonic dystrophy type 2-associated CCTG tetranucleotide repeats occur via double-strand break repair with distinct requirements for DNA helicases
Source: G3 (Bethesda). 2023 Nov 10;14(2):jkad257. doi: 10.1093/g3journal/jkad257 (PMC10849350; doi:10.1093/g3journal/jkad257)
Supplement: jkad257_Supplementary_Data [file jkad257_supplementary_data.zip › Supplementary_Figures_G3-2023-404472.pdf]

Papp *et al*
Supplementary Figure 1

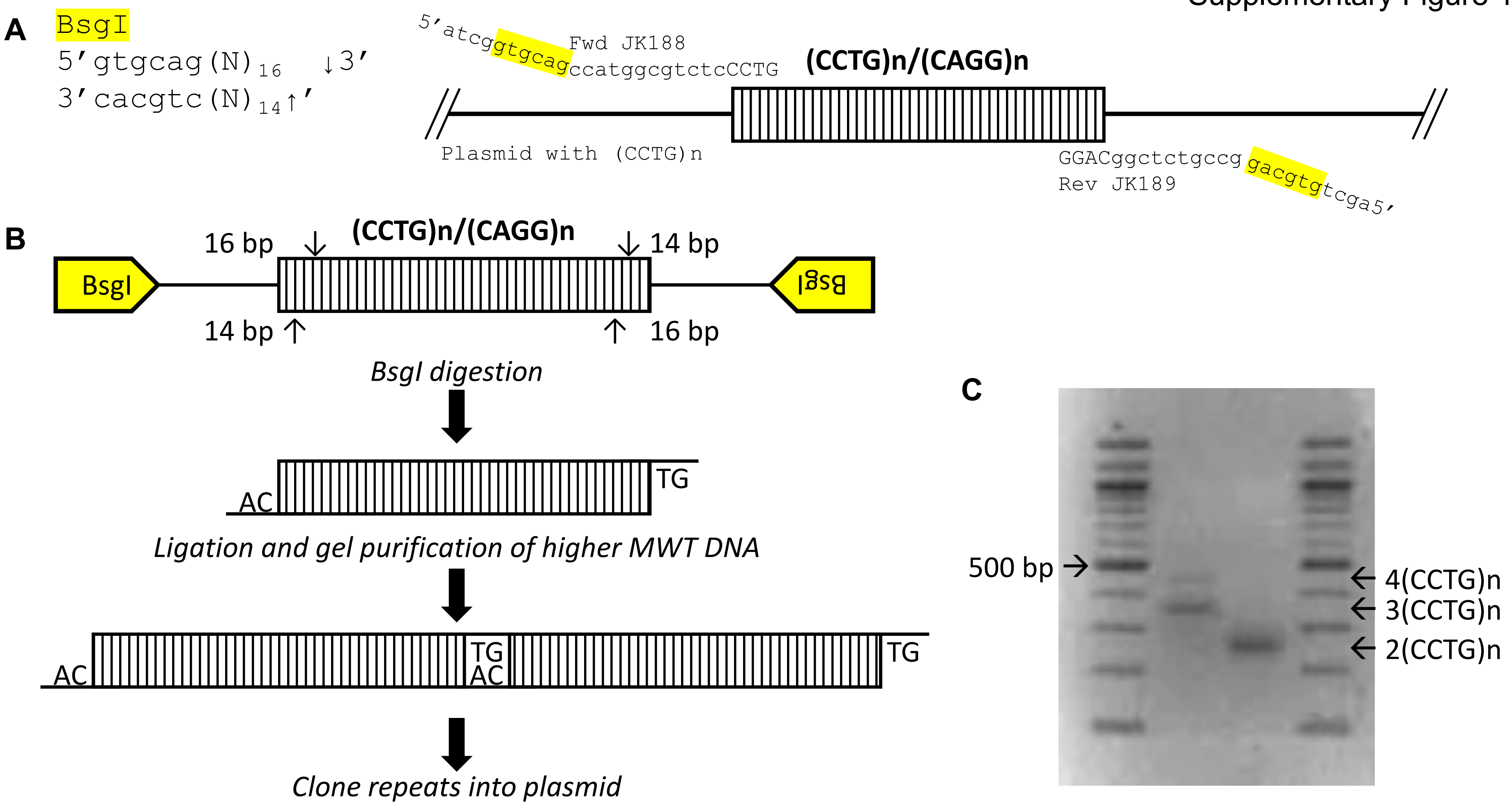

Supplementary Figure 1. Molecular cloning strategy to generate long (CCTG)n/(CAGG)n repeats.

(A) BsgI restriction enzyme recognizes non-palindromic DNA sequence and generates 3’ overhangs. Primers with BsgI sequences at 5’ end are used to generate (CCTG)n fragment with flanking, inverted BsgI sites.

(B) DNA fragment containing 5’ BsgI sites in an inverted orientation was generated using synthetic oligonucleotides and PCR. (CCTG)n/(CAGG)n repeats are positioned between the two inverted BsgI sites in such a way that pure repeats with 3’ overhangs TG and AC (part of the CCTG/CAGG unit) are generated upon BsgI digestion. These fragments will ligate in the head-to-tail direction only.

(C) DNA repeat fragments can be visualized on an agarose gel following purification.

A

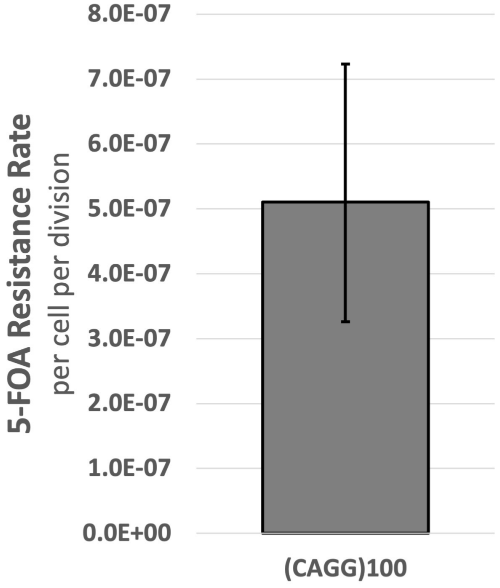

B

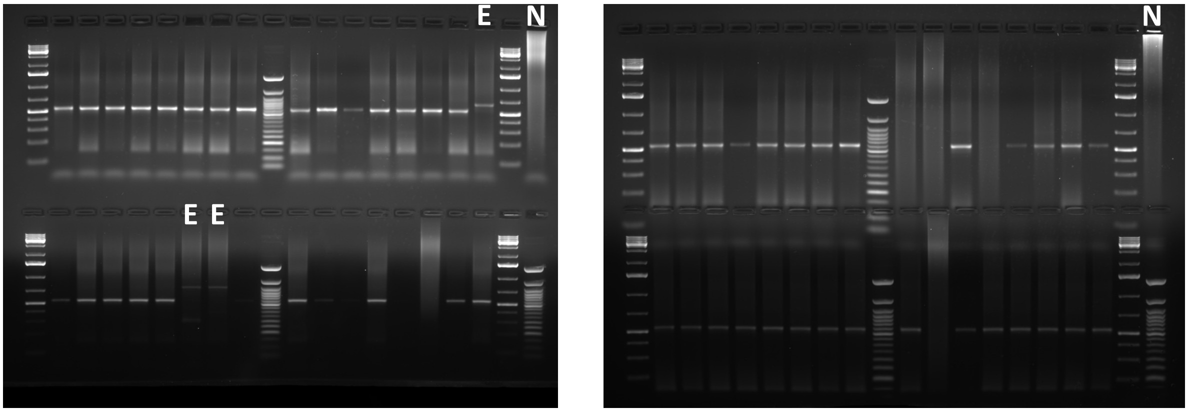

Supplementary Figure 2. 5-FOA resistant clones can be evaluated in the $(CAGG)_{100}$ strain.

(A) Rate of 5-FOA resistant clones for $(CAGG)_{100}$ strain is 5.1 x 10^{-7}, shown with 95% confidence intervals (3.3 x 10^{-7} – 7.2 x 10^{-7}). Rate is calculated with 12 independent cultures using the Ma-Sandri-Sarkar maximum-likelihood estimator with a correction for sampling and plating efficiency.

(B) PCR analysis of 5-FOA yeast clones. Expansions are designated with E, and no PCR product N.

A

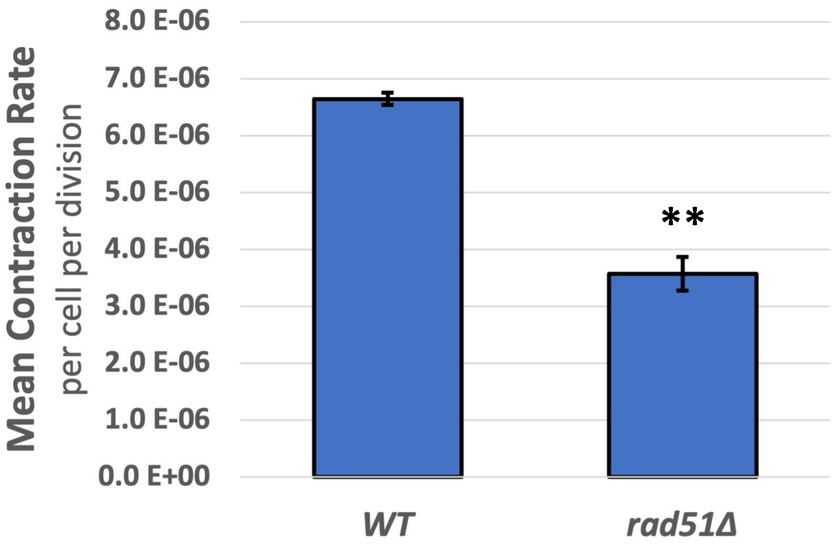

B

Trial #	Strain	Phenotype	#Cultures	ContRate	Lower C.I.	Upper C.I.	Fold-change		
1	YJK 168	WT	9	6.4031E-06	4.8589E-06	9.0998E-06			
	YJK 272	rad51Δ	11	2.9716E-06	2.0801E-06	3.9759E-06	0.4641		
2	YJK 168	WT	10	6.6996E-06	5.2132E-06	8.3198E-06			
	YJK 272	rad51Δ	12	3.5994E-06	2.5980E-06	4.7170E-06	0.5373		
3	YJK 168	WT	7	6.8238E-06	5.0055E-06	8.8429E-06			
	YJK 272	rad51Δ	10	4.1444E-06	3.0038E-06	5.4158E-06	0.6073		
						**AVG**	**StError**	**1-tailed, unpaired t-test**	
	YJK 168	WT	6.4031E-06	6.6996E-06	6.8238E-06	6.64E-06	1.0808E-07		
	YJK 272	rad51Δ	2.9716E-06	3.5994E-06	4.1444E-06	3.57E-06	2.9344E-07	0.00052457	

Supplementary Figure 3. Independent statistical analysis of large-scale CCTG contraction rate in *rad51Δ* mutant.

(A) Large-scale CCTG contraction rates were calculated with FluCalc, which uses the Ma-Sandri-Sarkar maximum likelihood estimator model with a correction for sampling and plating efficiency. Error bars indicate standard error from three independent experiments. Statistical significance was evaluated by unpaired t test (** $p<0.001$).
(B) Contraction rates, confidence numbers, and number of independent cultures per trial for comparing large-scale CCTG contraction rates in WT and *rad51Δ* mutant

Papp *et al*
Supplementary Figure 4

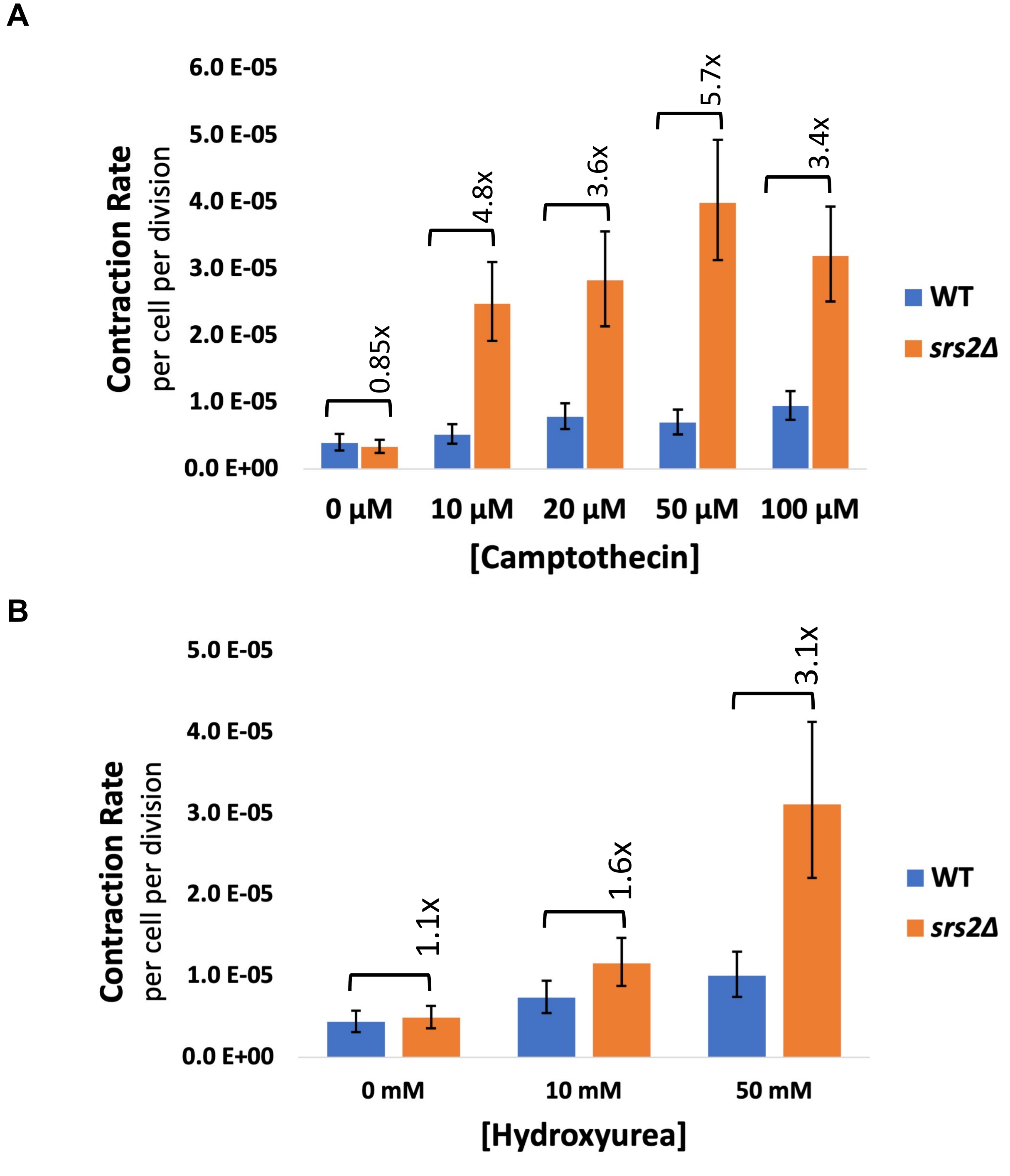

Supplementary Figure 4. Large-scale CCTG repeat contractions following replication stress in wild type and *srs2Δ* strains.

Rate of large-scale contraction for $(CCTG)_{100}$ strains, shown with 95% confidence intervals, following treatment with chemical stressors that affect DNA replication. Rate is calculated using the number of Ura+ clones starting with 12 independent cultures using the Ma-Sandri-Sarkar maximum-likelihood estimator with a correction for sampling and plating efficiency.

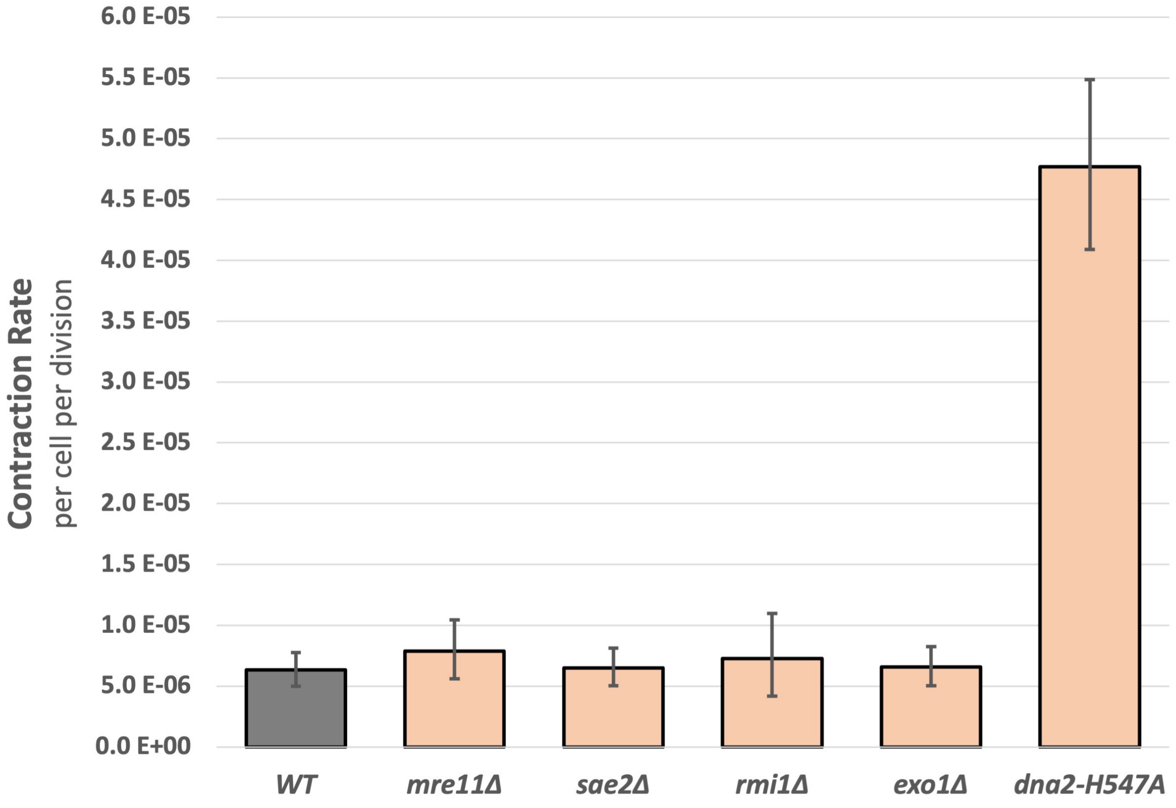

Supplementary Figure 5. Genetic analysis of large-scale CCTG repeat contractions.

Rate of large-scale contraction for $(CCTG)_{100}$ strains with mutations in genes involved in DNA resection, shown with 95% confidence intervals. Rate is calculated using the number of Ura+ clones in 12 independent cultures using the Ma-Sandri-Sarkar maximum-likelihood estimator with a correction for sampling and plating efficiency.

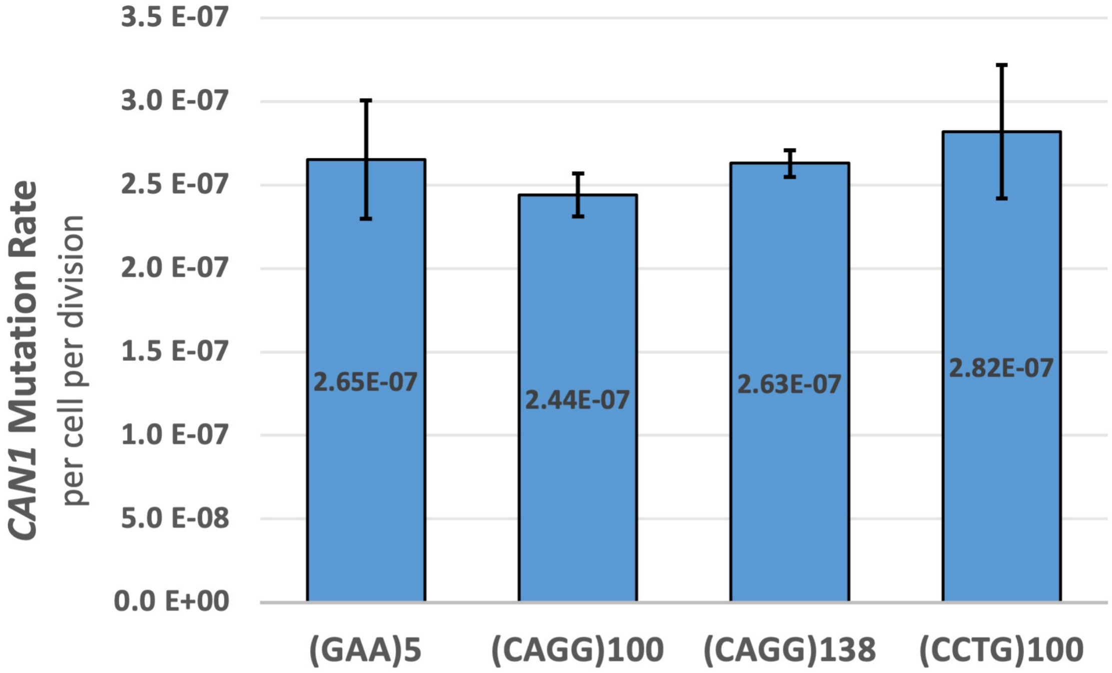

Supplementary Figure 6. CCTG/CAGG repeats do not affect *CAN1* mutation rate alone.

CAN1 mutation rates of $(GAA)_5$, $(CAGG)_{100}$, $(CAGG)_{138}$, and $(CCTG)_{100}$ from three-day incubation on selective media, shown with 95% confidence intervals. *CAN1* mutation rates were calculated with FluCalc, which uses the Ma-Sandri-Sarkar maximum likelihood estimator model with a correction for sampling and plating efficiency.

Papp *et al*
Supplementary Figure 7

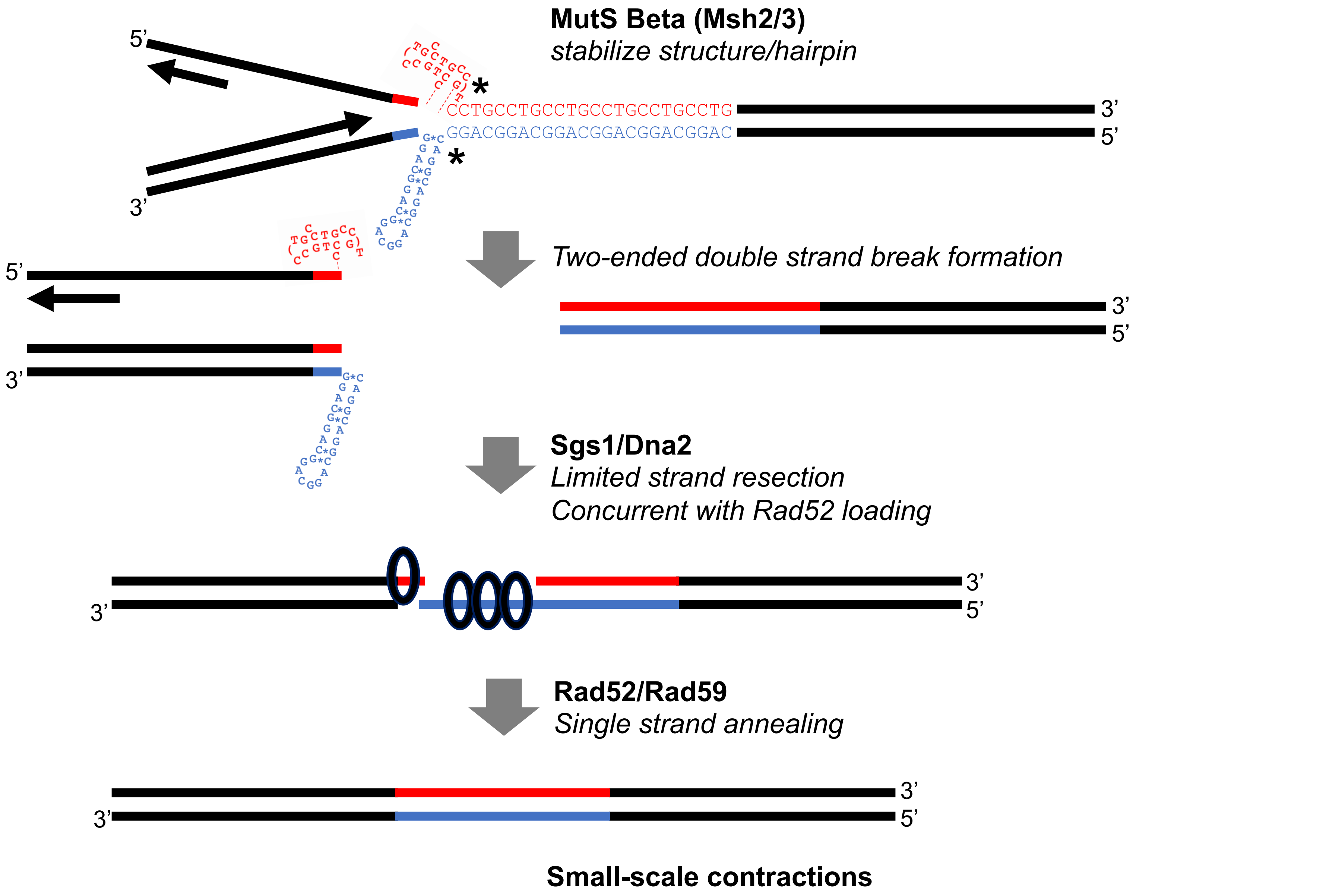

Supplementary Figure 7. Model of small-scale CCTG contractions in the presence of Sgs1 and Dna2 activity.
