# Supplementary material for: Massive contractions of myotonic dystrophy type 2-associated CCTG tetranucleotide repeats occur via double-strand break repair with distinct requirements for DNA helicases
Source: G3 (Bethesda). 2023 Nov 10;14(2):jkad257. doi: 10.1093/g3journal/jkad257 (PMC10849350; doi:10.1093/g3journal/jkad257)
Supplement: jkad257_Supplementary_Data [file jkad257_supplementary_data.zip › Supplementary_Text_and_Tables_G3-2023-404472.pdf]

## Supplementary Materials and Methods

*Fluctuation analysis to determine 5-FOA resistance rate in the $(CAGG)_{100}$ strain*

Strain YJK246 was grown as single colonies on rich media (YPD) agar plates supplemented with 50 µg/mL uracil (referred to as DU) for 72 hours at 30°C. Whole, individual colonies were suspended in 250 µL sterile water. To select for 5-FOA$^R$ clones, 100 µL of the cell suspension was directly plated on synthetic complete media with 0.1% 5-FOA (Toronto Research Chemicals). The cell suspension was serially diluted to plate on YPD media (100 µL of $10^{-5}$ dilution) for determination of total cell number. For each experiment, at least 12 independent colonies were analyzed from two strain isolates. Colonies growing on DU that had an initial contraction or expansion, assessed via repeat length PCR (JK213/547), were excluded from the analysis. All colonies on 5-FOA and YPD were counted at 72 hours. Rates and 95% confidence intervals were calculated using the Ma-Sandri-Sarkar maximum likelihood estimator (MSS-MLE) method with correction for plating efficiency determined as z-1/zln(z), where z is the fraction of the culture analyzed (*1*). The average number of viable cells grown on YPD (Nt) was used in all calculations. The web-hosted program FluCalc was used to perform this rate analysis (*2*).

**Supplementary Table 1. Primers used for CCTG/CAGG repeat cloning and RNA analysis**

| Name | Sequence | Notes |
|---|---|---|
| JK178_CCTG_ fwd | GTGccatggCGTCTC(CCTG)$_{21}$C | CCTG cloning (NcoI) |
| JK179_CAGG_ rev | GGTgcatgcCGTCTCGG(CAGG)$_{20}$CAG | CCTG cloning (SphI) |
| JK188_CCTG_BsgI_fwd | ATCGgtgcagCCATGGCGTCTCCCTG | CCTG cloning (BsgI) |
| JK189_CCTG_BsgI_rev | AGCTgtgcagGCCGTCTCGGCAGG | CCTG cloning (BsgI) |
| TrpS-F | TCGATTTCTGACTGGGTTGGAAG | Verify ChrIII integration (right) |
| 36a-R | TAGCATACGTGTAATGATAGACGATTTTC | Verify ChrIII integration (right) |
| A36b-F | ACGTGTACAGTTCTCTTTACATCATC | Verify ChrIII integration (left) |
| Ahead-R | AGTGTGATGGATATCTGCAGAA | Verify ChrIII integration (left) |
| JK15_URA3_fwd | CTAAAGGCATTATCCGCCAAG | Amplify repeats for sequencing |
| JK18_ACT_intron_rev | GGAGGTTATGGGAGAGTGAAAAATAG | Amplify repeats for sequencing |
| JK213_CCTG_fwd | GTCCTGTGGATCCTCTACGC | Verify repeat length |
| JK214_CCTG_rev | GAGGTTATGGGAGAGTGAAAAATAG | Verify repeat length |
| JK547_CCTG | GGTCCTCGCCGAAAATGAC | With JK213 to verify CAGG length |
| JK402_CCTG_fwd | CATCGGTGCAGCCATGGCGTCTCC | Verify repeat length |
| JK403_CCTG_rev | CTGTGCAGGCCGTCTCGGCAG | Verify repeat length |
| JK147_URA3_seq1 | ACAACATGGGTGGAGACAAAC | 5’ URA3 sequencing |
| JK148_URA3_seq2 | ATATCTGCAGAATTGGGACCGTGC | 5’ URA3 sequencing |
| JK149_URA3_seq3 | TCCCATAACCTCCTATATTGACTG | 3’ URA3 sequencing |
| JK150_URA3_seq4 | ACTCACGTGCTCAATAGTCACC | 3’ URA3 sequencing |
| JK354_CCTG_XhoI | agctctcgagCATTCCGACAGCATCGCCAGTC | CAGG orientation cloning |
| JK355_CCTG_NotI | agctgcggccgcATCATCGTCGCGCTCCAGCGAAAG | CAGG orientation cloning |
| | | |
| 5URA3_FWD | ATCCTAGTCCTGTTGCTGCCAA | 5’ unspliced *URA3* transcript |
| 5URA3_REV2 | AGAATTGGGACCGTGCAATTCTTC | 5’ unspliced *URA3* transcript |
| 3URA3_FWD2 | CTCGAGAGATTTCTCTTTTACC | 3’ unspliced *URA3* transcript |
| 3URA3REV | GATCCTGTAGAGACCACATCATC | 3’ unspliced *URA3* transcript |
| 5URA3_FWD | ATCCTAGTCCTGTTGCTGCCAA | Spliced *URA3* transcript |
| 3URA3_SPLICE_REV | AATTCTGCTAACATCAAAAGGcctc | Spliced *URA3* transcript |
| ACT1_fwd | AACAATGGATTCTGAGGTTGC | Control transcript |
| ACT1_rev | GAGTCTTTTTGACCCATACCG | Control transcript |

**Supplementary Table 2. Strains used in this study**

| Strain | Genotype | Background | Comments |
|---|---|---|---|
| CH1585 | *MATa, leu2-Δ1, trp1-Δ63, ura3–52, his3–200* | | Shishkin *et al*, 2009 |
| YJK237 | *ChrIII(75594-75641)::URA3-Int-TET644_TRP1* | | *URA3* (971 bp “long” intron) |
| YJK239 | *ChrIII(75594-75641)::URA3_TRP1* | | *URA3* (no intron) |
| YJK251 | *ChrIII(75594-75641)::URA3-Int-(CCTG)100* | | $(CCTG)_{100}$ 820 bp intron |
| YJK168 | *ChrIII(75594-75641)::URA3-Int-(CCTG)100* | | $(CCTG)_{100}$ 1047 bp intron |
| YJK246 | *ChrIII(75594-75641)::URA3-Int-(CAGG)100* | | $(CAGG)_{100}$ 1047 bp intron |
| YJK269 | *rad27Δ* | YJK168 | |
| YJK306 | *pol32::HphMX4* | YJK168 | |
| YJK272 | *rad51Δ* | YJK168 | |
| YJK274 | *rad52Δ* | YJK168 | |
| YJK309 | *rad51Δ rad52Δ* | YJK168 | |
| YJK273 | *rad51-Y388H* | YJK168 | |
| YJK275 | *rad52-Y33A* | YJK168 | |
| YJK313 | *rad59::HphMX4* | YJK168 | |
| YJK244 | *srs2Δ* | YJK168 | |
| YJK270 | *sgs1Δ* | YJK168 | |
| YJK271 | *sgs1-K706A* | YJK168 | |
| YJK314 | *rad51Δ sgs1Δ* | YJK168 | |
| YJK285 | *msh2Δ* | YJK168 | |
| YJK286 | *msh3Δ* | YJK168 | |
| YJK288 | *msh6Δ* | YJK168 | |
| YJK287 | *mlh1Δ* | YJK168 | |
| YJK289 | *mlh2Δ* | YJK168 | |
| YJK290 | *mlh3Δ* | YJK168 | |
| YJK291 | *pms1Δ* | YJK168 | |
| YJK307 | *sgs1Δ msh3Δ* | YJK168 | |
| YJK308 | *rad51Δ msh3Δ* | YJK168 | |
| YJK321 | *mre11::HphMX4* | YJK168 | |
| YJK330 | *sae2::HphMX4* | YJK168 | |
| YJK322 | *rmi1::HphMX4* | YJK168 | |
| YJK323 | *exo1::HphMX4* | YJK168 | |
| YJK315 | *dna2-H547A* | YJK168 | |
| KT119/120 | *MATa, his7-2, leu2-3,112, trp1-Δ, ura3-Δ, lys2-Δ, ade2-Δ, bar1-Δ, sfa1-Δ, cup1-1-Δ, yhr054c-Δ, cup1-2-Δ, lys2::kanMXURA3, ADE2, CUP1, SFA1* | | Kim *et al*, 2008 |
| HMK1/2 | *lys2::*$(GAA)_{5}$ | KT119/120 | Kim *et al*, 2008 |
| HMK21/22 | *lys2::*$(GAA)_{230}$ | KT119/120 | Kim *et al*, 2008 |
| YJK300-2 | *lys2::*$(CAGG)_{100}$ | KT119 | |
| YJK302-5 | *lys2::*$(CAGG)_{100}$ | KT120 | |
| YJK302-3 | *lys2::*$(CAGG)_{138}$ | KT120 | |
| YJK301-8 | *lys2::*$(CCTG)_{100}$ | KT120 | |

**Supplementary Table 3. Primers used for genetic analysis and mutant strain construction**

| Name | Sequence | Notes |
|---|---|---|
| JK419_RAD27_gRNA1_fwd | AAAGCGGTCTTCAAGAAGGGGTTTT | CRISPR plasmid |
| JK420_RAD27_gRNA1_rev | CCCTTCTTGAAGACCGCTTTGATCA | CRISPR plasmid |
| JK423_RAD27_donor | ATGGGTATTAAAGGTTTGAATGCAATTATATCGGAACATGTTCCCAAATTGAACAAAAATAAGAATAAAGTCACAAAGGGAAGAAGATGA | KO repair template |
| JK430_Rad27_Seq_F | TAACATCGCGCAAATGAAGG | Verify KO |
| JK431_Rad27_Seq_R | AAAATTCCACGTTCAAGTTCCC | Verify KO |
| POL32_pRS_F | CACATTAACTAACAACCAGAAATAGGCTTTAGTTAACTCAATCGGTAATTGGTGTCGGGGCTGGCTTAA | Direct replacement PCR |
| POL32_pRS_R | TGACATTTGTATTATACATTACATCACAATTAGTAATGGAAAGTGTTTGGGTTTACAATTTCCTGATGCGGTA | Direct replacement PCR |
| JK225_pol32_fwd | tttccactacggtgtaactttcc | Check integration |
| JK183_hygRleft_rev | ACAGTCACATCATGCCCCTG | Check integration |
| Pol32_in_F | gaccacgccagaagaaacaa | Check absent in KO |
| Pol32_in_R | gctgtcgtttccaacaagtc | Check absent in KO |
| JK472_RAD51_gRNA_fwd | TGACTCACCTTGCTTACCAGGTTTT | CRISPR plasmid |
| JK473_RAD51_gRNA_rev | CTGGTAAGCAAGGTGAGTCAGATCA | CRISPR plasmid |
| JK474_RAD51_donor_KO | ATGTCTCAAGTTCAAGAACAACATATATCAGAGTCACAGCTTCAGATCTATGAAGATGGTGTTGGTGACCCCAGAGAAGAAGACGAGTAG | KO repair template |
| JK475_RAD51_donor_Y388H | GTTGTTGACTCACCTTGCTTACCAGAAGCTGAATGTGTGTTCGCGATCCATGAAGATGGTGTTGGTGACCCCAGAGAAGAAGACGAGTAG | Y338H repair template |
| JK498_RAD51_gene_fwd | TCGCGGCTGGTGAATAACGCTGC | Verify KO |
| JK499_RAD51_gene_rev | AAGACCGCAGTAGGGTTGCGAGG | Verify KO |
| JK500_RAD51_Y388H_fwd | TCCATTGGATATTGGTGGCGGTGAAGG | Verify mutation |
| JK476_RAD52_gRNA_fwd | ATATAATGGCTGGTCTACGGGTTTT | CRISPR plasmid |
| JK477_RAD52_gRNA_rev | CCGTAGACCAGCCATTATATGATCA | CRISPR plasmid |
| JK478_RAD52_donor_KO | ATGAATGAAATTATGGATATGGATGAGAAGAAGCCCGTTTTCGGTGGAAGACCAAAGATCAATCCCCTGCATGCACGCAAGCCTACTTGA | KO repair template |
| JK479_RAD52_donor_Y33A | TTCGGTAACCATTCCGAGGACATACAGACCAAACTTGACAAGAAATTAGGACCTGAGGCCATCTCCAAGAGAGTTGGGTTTGGAACAAGC | Y33A repair template |
| JK501_RAD52_gene_fwd | CTGGGAAGGTCGGGAACAGGCATAAG | Verify KO |
| JK502_RAD52_gene_rev | AACGGTGAGTGTGGCAACGCCAG | Verify KO |
| JK503_RAD52_Y33A_fwd | GCGGCAGGTTTCCGTCTTTCGTTCTC | Verify mutation |
| Rad59-pAG-F | GGTTACGTAGAGGAGAAGAGCATATTTCAGGATAAACAGACAAAATAATGCAGCTGAAGCTTCGTACGC | Direct replacement PCR |
| Rad59-pAG-R | GTGAAAATTATGACTTTTTATCAAGCAAAATAAATTTGCTACTTGTGCCCCATAGGCCACTAGTGGATCTG | Direct replacement PCR |
| Rad59-dnstr-R | CCTTCGTTACCTTGGAATGG | Check integration |
| Rad59-upstr-F | GCAAGGGCAGATATGATAGG | Check integration |
| Rad59-F | TTCGACTACATACGGCACAG | Check absent in KO |
| Rad59-R | GCTTGCTATTAGTCGCTGAC | Check absent in KO |
| JK376_SRS2_gRNA1_fwd | GAAATCATCATCTTTCGAGGGTTTT | CRISPR plasmid |
| JK377_SRS2_gRNA1_rev | CCTCGAAAGATGATGATTTCGATCA | CRISPR plasmid |
| JK380_SRS2_donor | ATGTCGTCGAACAATGATCTTTGGTTGCATTTAGTATCCCAGTTAAAAAAGTCAAAATTAAACAACGGTGAAATCATAGTCATCGATTAG | KO repair template |
| JK381_SRS2_chk_fwd | acacaaccactgcttcgtattg | Verify KO |
| JK382_SRS2_chk_rev | cttcgacttgggactattggac | Verify KO |

| | | |
|---|---|---|
| JK230_MRE11_SP | ttaagagaatgcagacaattgacgcaagttgtacctgctcagatccgataaaactcgactCAGCTGAAGCTTCGTACGC | Direct replacement PCR |
| JK231_MRE11_ASP | tcgcgaaggcaagcccttggttataaataggatataatataatatagggatcaagtacaaCATAGGCCACTAGTGGATCTG | Direct replacement PCR |
| JK232_MRE11_fwd | ccaatcatttcgaccgtcactc | Check integration |
| JK233_MRE11_rev | cacaaggggacggttaatgagg | Check integration |
| JK254_MRE11_int_fwd | TGGATATACTTCATGCGACTGG | Check absent in KO |
| JK255_MRE11_int_rev | ATGACCCCATATCACCATATCC | Check absent in KO |
| JK637_SAE2_SP | cgttcacatacctgcatttccatccatgctgtaagccattaggtgtttgtatgtgagatgCAGCTGAAGCTTCGTACGC | Direct replacement PCR |
| JK638_SAE2_ASP | tcaaccataccaaaaaaaatgtatttgaagtaatgaataaagaatgatgatcgctggcgtCATAGGCCACTAGTGGATCTG | Direct replacement PCR |
| JK639_SAE2_fwd | atgccattttctaagcgcgttc | Check integration |
| JK640_SAE2_rev | tttctgctttaccaactgctgc | Check integration |
| JK641_SAE2_int_fwd | TGCTCCTCAACAATCCTCTCAG | Check absent in KO |
| JK642_SAE2_int_rev | CAGGGTTCTCGAGCTCTATTCC | Check absent in KO |
| JK278_RMI1_SP | agggagtactgagaagaaggccaaaatactgcagagcatacatactaccgagtggatggaCAGCTGAAGCTTCGTACGC | Direct replacement PCR |
| JK279_RMI1_ASP | atgtaatatttaacgtatacataacgggcagaacgcccttctaagaagaagaggcatatgCATAGGCCACTAGTGGATCTG | Direct replacement PCR |
| JK280_RMI1_fwd | gcacatatgctccaaccaatac | Check integration |
| JK281_RMI1_rev | cagctctagcgttgtaacatgg | Check integration |
| JK282_RMI1_int_fwd | aaatggcgctaagaagtacgag | Check absent in KO |
| JK283_RMI1_int_rev | gccgagaaaaatgacttgtgag | Check absent in KO |
| JK605_EXO1_SP | gaaaggcgtagaaaggaATGGGTATCCAAGGTCTTCTTCCTCAGTTAAAGCCCATACAGAcagctgaagcttcgtacgc | Direct replacement PCR |
| JK606_EXO1_ASP | atttcgacgagattttcatttgaaaaatatacctccgatatgaaacgtgcagtacttaaccataggccactagtggatctg | Direct replacement PCR |
| JK607_EXO1_fwd | GTATTACGTCCAAACTAAGTTCGCG | Check integration |
| JK608_EXO1_rev | GACCGCTAGCGGCTTGATTAG | Check integration |
| JK609_EXO1_int_fwd | CAGCGGGAGGGAAAACTGA | Check absent in KO |
| JK610_EXO1_int_rev | CTCTGTTGGCTAGAGGTTGGTG | Check absent in KO |
| DNA2_H547A_pRCCN_F | TATTGCCTAAAGTCATGACAGTTTTAGAGCTAGAAA TAGCAAGTTAAAATAAGG | Amplify pRCC-N template |
| DNA2_H547A_pRCCN_R | TGTCATGACTTTAGGCAATACGATCATTTATCTTTCA CTGCGGAG | Amplify pRCC-N template |
| DNA2_H547A_mut_F | AAGACGTTCAATTCTGCAAATGCAATTTCAAGATCC CCGCGGAGAACCAAGTCTTGTCA TGACTTT AGGCAA TATCGTAGCCGAGTTATTGCAAGACTC | Amplify repair template |
| DNA2_R | CTTCAATATCAATCACATTGGATATAGAAATTGGCT GTGTTCTTCTTGTTCCGGAA | Amplify repair template |
| DNA2_seq_R | CCTTGAACCTCGT A TGAAACGCTTC | Sanger sequencing |
| DNA2_seq_F | GCTAACGACAACCTGTTGGTGC | Sanger sequencing |
| JK468_SGS1_gRNA_fwd | GTTCTTATGCCAACAGGGGGGTTTT | CRISPR plasmid |
| JK469_SGS1_gRNA_rev | CCCCCTGTTGGCATAAGAACGATCA | CRISPR plasmid |
| JK470_SGS1_donor_KO | ATGGTGACGAAGCCGTCACATAACTTAAGAAGGGAGCACAAATGGCGAGGTTTTAGAAATTACCGAGGTCACTACAGAGGAAGAAAGTGA | KO repair template |
| JK471_SGS1_donor_K706A | TGCAAGGTAAGGATGTTTTTGTTCTTATGCCAACAGGGGGAGGTGCCTCTCTTTGCTATCAACTTCCTGCAGTGGTGAAATCGGGTAAAA | K706A repair template |

| | | |
|---|---|---|
| JK494_SGS1_gene_fwd | TTCCTCATGGTGGTGGTGGTCCAGC | Verify KO |
| JK495_SGS1_gene_rev | AGGTGGACCCGTGACATTCGCAG | Verify KO |
| JK496_SGS1_K706A_fwd | AGTTTGTCGTCTTTGCTCGGCAGTACC | Verify mutation |
| JK497_SGS1_K706A_rev | GCATGAGTTTCTCCTTTGGCCGTCAACAC | Verify mutation |
| JK443_MSH2_gRNA1_fwd | TGATACCACTGCATACAAGGGTTTT | CRISPR plasmid |
| JK444_MSH2_gRNA1_rev | CCTTGTATGCAGTGGTATCAGATCA | CRISPR plasmid |
| JK445_MSH2_donor | ATGTCCTCCACTAGGCCAGAGCTAAAATTCTCTGATGTATCAGAG GAAAACGATAATTACCTGAAATATATAAAAGCCTTGTTGTTATAA | KO repair template |
| JK486_MSH2_fwd | CCATGTGTCTTCTTCTGACGAGC | Verify KO |
| JK487_MSH2_rev | CTCGCCGATGATGGACTGAATAC | Verify KO |
| JK446_MSH3_gRNA1_fwd | TGACGTACATCAGGGAAAGGGTTTT | CRISPR plasmid |
| JK447_MSH3_gRNA1_rev | CCTTTCCCTGATGTACGTCAGATCA | CRISPR plasmid |
| JK448_MSH3_donor | ATGGCGGGACAACCCACAATAAGCAGGTTTTTCAAGAAGGCGGT AGCAACGGATAAACTCGCGAAATTACTATCATTGGATATCCACTG A | KO repair template |
| JK484_MSH3_fwd | AAATCACGGTATGTGGCCGATAG | Verify KO |
| JK485_MSH3_rev | ATCCATCCAAGCAACGGGATC | Verify KO |
| JK449_MSH6_gRNA1_fwd | TTTGACTTAAAGATTGCAGGGTTTT | CRISPR plasmid |
| JK450_MSH6_gRNA1_rev | CCTGCAATCTTTAAGTCAAAGATCA | CRISPR plasmid |
| JK451_MSH6_donor | ATGGCCCCAGCTACCCCTAAAACTTCTAAGACTGCACACTTCGAAG TTTTGAAAAGTCTATTTAGTATAATTGATGATTTACAATCCTAA | KO repair template |
| JK535_MSH6_fwd_v2 | TGGAGCCTTCTTTGCTAACAG | Verify KO |
| JK536_MSH6_rev_v2 | AAGTCAGTTCATTCCGGTGAG | Verify KO |
| JK458_MLH1_gRNA1_fwd | AAATGTAAGCCACTCCCAAGGTTTT | CRISPR plasmid |
| JK459_MLH1_gRNA1_rev | CTTGGGAGTGGCTTACATTTGATCA | CRISPR plasmid |
| JK462_MLH1_donor | ATGTCTCTCAGAATAAAAGCACTTGATGCATCAGTGGTTAACAAA ATAGCCAACCTTCCAGATCTATACAAAGTTTTTGAGAGGTGTTAA | KO repair template |
| JK480_MLH1_fwd | CAAAGATAGTGTAGGAGGCGCTG | Verify KO |
| JK481_MLH1_rev | TTTGTGGAATCGGCTGCTGTC | Verify KO |
| JK516_MLH2_gRNA1_fwd | GATGTAGATTCCACTACGGGGTTTT | CRISPR plasmid |
| JK517_MLH2_gRNA1_rev | CCCGTAGTGGAATCTACATCGATCA | CRISPR plasmid |
| JK518_MLH2_donor | ATGACTATACATCAGTTATCTCCAGAATCCCAATGGAAGATTGTTG CCACGACAAATGAATGGTGCCTCTTCACGCCAGACAGCCCCTGA | KO repair template |
| JK529_MLH2_fwd | TCATCTCGGTTTGAGGAACAG | Verify KO |
| JK530_MLH2_rev | GTGCGGTTACCATGAGTTACA | Verify KO |
| JK519_MLH3_gRNA1_fwd | CGGTTGATAAATTATATTGGGTTTT | CRISPR plasmid |
| JK520_MLH3_gRNA1_rev | CCAATATAATTTATCAACCGGATCA | CRISPR plasmid |
| JK521_MLH3_donor | ATGAGCCAGCATATTAGGAAATTAGATTCTGATGTTTCTGAAAGG CCCACGGGAGACCTTCTATGGTACCCATTGCAGAATTGAAGTAA | KO repair template |
| JK531_MLH3_fwd | AACCAGCGAGGCTTTCAAGG | Verify KO |
| JK532_MLH3_rev | GCAGGCGACAAACCTTGTT | Verify KO |
| JK522_PMS1_gRNA1_fwd | TCCTCAGTATTCGGTGCAGGGTTTT | CRISPR plasmid |
| JK523_PMS1_gRNA1_rev | CCTGCACCGAATACTGAGGAGATCA | CRISPR plasmid |
| JK524_PMS1_donor | ATGACACAAATTCATCAGATAAACGATATAGATGTTCATCGAATT GAATTACGTGATTGGAGCTCATTTTCGAAGGATTACGAAATATGA | KO repair template |
| JK533_PMS1_fwd | TATGTCCAGCAGTTTCCATCAG | Verify KO |
| JK534_PMS1_rev | GGTCTTGTATCTTTGGCAAGTG | Verify KO |

**Supplementary Table 4. Primers used for GCR strain construction**

| Name | Sequence | Notes |
|---|---|---|
| JK510_GCR_CCTG_fwd | AAAGAACTTTGGCTTGCCGACGGCGGCTAAGCTCATAACATTGATAGTTGAAATAACATTCATCGGTGCAGCCATGGCGTCTCC | GCR strain construction |
| JK511_GCR_CCTG_rev | TGATATAATTATCCATAATGGTGCGTTAGTTCACTGGGTTTATCCATATGCCAAATTGAGCTGTGCAGGCCGTCTCGGCAG | GCR strain construction |
| JK512_GCR_CCTG_fwd | AAAGAACTTTGGCTTGCCGACGGCGGCTAAGCTCATAACATTGATAGTTGAAATAACATTCTGTGCAGGCCGTCTCGGCAG | GCR strain construction |
| JK513_GCR_CCTG_rev | TGATATAATTATCCATAATGGTGCGTTAGTTCACTGGGTTTATCCATATGCCAAATTGAGCATCGGTGCAGCCATGGCGTCTCC | GCR strain construction |
| JK552_kanMX_gRNA1_fwd | gccgcgattaaattccaacaGTTTT | CRISPR plasmid |
| JK553_kanMX_gRNA1_rev | tgttggaatttaatcgcggcGATCA | CRISPR plasmid |
| JK514_LYS2_fwd | tgattctaaaatgcctggcttc | Verify repeat length |
| JK515_LYS2_rev | aaaaggcaggtatcacctatgg | Verify repeat length |

**Supplementary Table 5. Contraction rates and 95% confidence intervals as determined by the Ma-Sandri-Sarkar maximum likelihood estimator (MSS-MLE) method**

| Strain | Description | Contraction Rate | Lower C.I. | Upper C.I. | Fold change | Fold DECREASE |
|---|---|---|---|---|---|---|
| YJK 168 | *WT* | 6.40E-06 | 4.86E-06 | 9.10E-06 | | |
| YJK 269 | *rad27Δ* | 5.87E-06 | 4.53E-06 | 7.34E-06 | 0.917 | |
| YJK 306 | *pol32Δ* | 9.95E-06 | 7.79E-06 | 1.23E-05 | 1.554 | |
| YJK 272 | *rad51Δ* | 2.97E-06 | 2.08E-06 | 3.98E-06 | 0.464 | 2.155 |
| YJK 274 | *rad52Δ* | 2.77E-06 | 1.71E-06 | 4.00E-06 | 0.432 | 2.315 |
| YJK 309 | *rad51/rad52Δ* | 3.91E-06 | 2.74E-06 | 5.22E-06 | 0.610 | 1.638 |
| YJK 273 | *rad51-Y388H* | 3.75E-06 | 2.11E-06 | 5.72E-06 | 0.586 | 1.706 |
| YJK 275 | *rad52-Y33A* | 7.00E-06 | 5.19E-06 | 9.01E-06 | 1.094 | |
| YJK 313 | *rad59Δ* | 5.79E-06 | 4.43E-06 | 7.28E-06 | 0.904 | |
| YJK 244 | *srs2Δ* | 6.17E-06 | 4.49E-06 | 8.03E-06 | 0.963 | |
| YJK 270 | *sgs1Δ* | 1.13E-06 | 6.98E-07 | 1.64E-06 | 0.177 | 5.656 |
| YJK 271 | *sgs1-K706A* | 1.17E-06 | 7.49E-07 | 1.65E-06 | 0.182 | 5.487 |
| YJK 314 | *rad51/sgs1Δ* | 5.91E-07 | 3.05E-07 | 9.43E-07 | 0.092 | 10.830 |
| YJK 285 | *msh2Δ* | 5.79E-06 | 4.23E-06 | 7.52E-06 | 0.904 | |
| YJK 286 | *msh3Δ* | 9.15E-07 | 5.17E-07 | 1.39E-06 | 0.143 | 6.999 |
| YJK 288 | *msh6Δ* | 9.96E-06 | 7.52E-06 | 1.26E-05 | 1.555 | |
| YJK 287 | *mlh1Δ* | 6.37E-06 | 4.43E-06 | 8.56E-06 | 0.995 | |
| YJK 289 | *mlh2Δ* | 5.78E-06 | 4.38E-06 | 7.31E-06 | 0.902 | |
| YJK 290 | *mlh3Δ* | 6.94E-06 | 5.29E-06 | 8.74E-06 | 1.084 | |
| YJK 291 | *pms1Δ* | 1.14E-05 | 8.79E-06 | 1.42E-05 | 1.779 | |
| YJK 307 | *sgs1/msh3Δ* | 5.83E-07 | 3.17E-07 | 9.05E-07 | 0.091 | 10.978 |
| YJK 308 | *rad51/msh3Δ* | 3.24E-06 | 2.32E-06 | 4.27E-06 | 0.506 | 1.976 |
| **Supplementary Figure 5** | | | | | | |
| YJK 321 | *mre11Δ* | 7.90E-06 | 5.61E-06 | 1.05E-05 | 1.248 | |
| YJK 323 | *exo1Δ* | 6.57E-06 | 5.04E-06 | 8.24E-06 | 1.038 | |
| YJK 322 | *rmi1Δ* | 7.28E-06 | 4.17E-06 | 1.10E-05 | 1.150 | |
| YJK 330 | *sae2Δ* | 6.51E-06 | 5.02E-06 | 8.14E-06 | 1.029 | |
| YJK 315 | *dna2-H547A* | 4.77E-05 | 4.10E-05 | 5.49E-05 | 7.540 | |

**Supplementary Table 6. Contraction rates and 95% confidence intervals as determined by the Ma-Sandri-Sarkar maximum likelihood estimator (MSS-MLE) method for camptothecin (CPT) and hydroxyurea (HU) treatments**

| Strain | Description | Drug Treatment | Contraction Rate | Lower C.I. | Upper C.I. | Fold change compared to 0 (same strain) * | Fold change compared to WT (same dose) |
|---|---|---|---|---|---|---|---|
| YJK 168 | WT | 0 uM CPT | 3.96E-06 | 2.80E-06 | 5.25E-06 | | |
| YJK 168 | WT | 10 uM CPT | 5.20E-06 | 3.81E-06 | 6.74E-06 | 1.313 | |
| YJK 168 | WT | 20 uM CPT | 7.86E-06 | 6.04E-06 | 9.84E-06 | 1.985 * | |
| YJK 168 | WT | 50 uM CPT | 7.01E-06 | 5.24E-06 | 8.96E-06 | 1.770 | |
| YJK 168 | WT | 100 uM CPT | 9.48E-06 | 7.41E-06 | 1.17E-05 | 2.394 * | |
| YJK 244 | srs2Δ | 0 uM CPT | 3.38E-06 | 2.47E-06 | 4.39E-06 | | 0.854 |
| YJK 244 | srs2Δ | 10 uM CPT | 2.48E-05 | 1.92E-05 | 3.10E-05 | 7.337 * | 4.769 |
| YJK 244 | srs2Δ | 20 uM CPT | 2.83E-05 | 2.14E-05 | 3.56E-05 | 8.373 * | 3.601 |
| YJK 244 | srs2Δ | 50 uM CPT | 3.99E-05 | 3.13E-05 | 4.93E-05 | 11.805 * | 5.692 |
| YJK 244 | srs2Δ | 100 uM CPT | 3.19E-05 | 2.51E-05 | 3.93E-05 | 9.438 * | 3.365 |
| | | | | | | | |
| YJK 168 | WT | 0 mM HU | 4.32E-06 | 3.07E-06 | 5.72E-06 | | |
| YJK 168 | WT | 10 mM HU | 7.33E-06 | 5.44E-06 | 9.41E-06 | 1.697 | |
| YJK 168 | WT | 50 mM HU | 1.01E-05 | 7.40E-06 | 1.30E-05 | 2.329 * | |
| YJK 244 | srs2Δ | 0 mM HU | 4.87E-06 | 3.56E-06 | 6.33E-06 | | 1.128 |
| YJK 244 | srs2Δ | 10 mM HU | 1.16E-05 | 8.76E-06 | 1.47E-05 | 2.379 * | 1.581 |
| YJK 244 | srs2Δ | 50 mM HU | 3.11E-05 | 2.21E-05 | 4.13E-05 | 6.393 * | 3.095 |

* Significance, as described in Materials and Methods

## Supplementary Literature Cited

1. W. A. Rosche, P. L. Foster, Determining mutation rates in bacterial populations. *Methods* **20**, 4-17 (2000).
2. E. A. Radchenko, R. J. McGinty, A. Y. Aksenova, A. J. Neil, S. M. Mirkin, Quantitative Analysis of the Rates for Repeat-Mediated Genome Instability in a Yeast Experimental System. *Methods Mol Biol* **1672**, 421-438 (2018).
